# Quality of information in news media reports about the effects of health interventions: Systematic review and meta-analyses

**DOI:** 10.12688/f1000research.52894.2

**Published:** 2022-01-31

**Authors:** Matt Oxman, Lillebeth Larun, Giordano Pérez Gaxiola, Dima Alsaid, Anila Qasim, Christopher James Rose, Karin Bischoff, Andrew David Oxman

**Affiliations:** 1 Centre for Informed Health Choices, Norwegian Institute of Public Health, Oslo, Norway; 2 Faculty of Health Sciences, Oslo Metropolitan University, Oslo, Norway; 3 Division for Health Services, Norwegian Institute of Public Health, Oslo, Norway; 4 Cochrane Associated Centre and Evidence‐based Medicine Department, Sinaloa's Pediatric Hospital, Culiacan, Mexico; 5 Institute for Evidence in Medicine (for Cochrane Germany Foundation), Faculty of Medicine and Medical Center, University of Freiburg, Freiburg, Germany; 6 Department of Health Research Methods, Evidence and Impact, McMaster University, Hamilton, Ontario, Canada

**Keywords:** news, news media, news reports, health news, systematic review, meta-analysis, infodemic

## Abstract

Background

Many studies have assessed the quality of news reports about the effects of health interventions, but there has been no systematic review of such studies or meta-analysis of their results. We aimed to fill this gap (PROSPERO ID: CRD42018095032).

Methods

We included studies that used at least one explicit, prespecified and generic criterion to assess the quality of news reports in print, broadcast, or online news media, and specified the sampling frame, and the selection criteria and technique. We assessed criteria individually for inclusion in the meta-analyses, excluding ineligible criteria and criteria with inadequately reported results. We mapped and grouped criteria to facilitate evidence synthesis. Where possible, we extracted the proportion of news reports meeting the included criterion. We performed meta-analyses using a random effects model to estimate such proportions for individual criteria and some criteria groups, and to characterise heterogeneity across studies.

Results

We included 44 primary studies in the review, and 18 studies and 108 quality criteria in the meta-analyses. Many news reports gave an unbalanced and oversimplified picture of the potential consequences of interventions. A limited number mention or adequately address conflicts of interest (22%; 95% CI 7%-49%) (low certainty), alternative interventions (36%; 95% CI 26%-47%) (moderate certainty), potential harms (40%; 95% CI 23%-61%) (low certainty), or costs (18%; 95% CI 12%-28%) (moderate certainty), or quantify effects (53%; 95% CI 36%-69%) (low certainty) or report absolute effects (17%; 95% CI 4%-49%) (low certainty).

Discussion

There is room for improving health news, but it is logically more important to improve the public’s ability to critically appraise health information and make judgements for themselves.

## Introduction

For decades, researchers have studied and criticised the quality of health information in the mass media. In 1972, The New England Journal of Medicine—the “most prestigious” general medical journal in the world ^1^—published an “examination” of health information on American television (TV). ^2^ The paper ended in reproach: “The potential of television to inform, instruct and educate should not continue to be wasted and abused, but rather should be used as an integral part of a plan designed to deliver better health care.”

Misleading health information in the mass media continues to be a hot topic. In 2020, the World Health Organization reported that the Coronavirus disease (COVID-19) had been accompanied by an “infodemic”: “an over-abundance of information—some accurate and some not—that makes it hard for people to find trustworthy sources and reliable guidance when they need it.”. ^3^

Many studies of health news have focused on “traditional” news media, including print, broadcast, and news websites. With the advent of social media, traditional news media have increased competition as a source of lay health information. However, people still get health information from the latter, directly or via social media. Chew and Eysenbach found that about 60% of more than 5000 tweets about the H1N1 outbreak in 2009 included links and about a quarter of those links were to news websites, the most common destination. ^4^ In comparison, less than 5% of links were to government and public health agencies or to other social media, combined.

Many of the studies focusing on news media have further focused on news reports about the effects of health interventions. This includes “modern” medicine (aka. “academic”, “conventional” or “Western” medicine); “alternative” medicine (aka. “complementary”, “traditional” or “natural” medicine); screening; surgery; devices; diet; exercise; lifestyle interventions; and health systems and policies. We were unable to find a systematic review of such studies (see extended data - S1, S2 and S3 Files ^105^). ^5^ We then aimed to fill this gap, providing meta-analytical estimates of the quality of news reports about the effects of health interventions, and potentially informing further and related research.

The first part of this study was submitted by the first author, MO, to the University of Oxford as his dissertation, in partial fulfilment of the requirement for the award of Master of Science (MSc) in Evidence-Based Health Care, under the title “Criteria used to measure the quality of news media report about the effects of health interventions: Systematic review” (see extended data - S4 File ^105^).

The study is related to the Informed Health Choices (IHC) project, in which MO, GG, CR and AO have been involved. The project goal is to help people think critically about information about the effects of health interventions, as well as choices about health interventions, so they avoid waste and unnecessary suffering. A premiss for the project is that there is a large amount of unreliable information about the effects of health intervention. This review helped test this premiss, and takes advantage of the IHC Key Concepts framework, which MO and AO have helped develop.

### Objectives

Primary:

• To assess the quality of information in news media reports about the effects of health interventions.

Secondary:

• To assess, map and group criteria used to measure the quality of information in news media reports about the effects of health interventions.

## Methods

This review was registered in the International Prospective Register of Systematic Reviews ( PROSPERO) (ID: CRD42018095032). The protocol was published on the IHC project website as “Quality of news media reports about the effects and costs of health interventions: Systematic review protocol” (see extended data - S1, S2 and S3 Files ^105^). ^5^ Appendixes to the protocol were published in a separate compressed folder, on the same website. A Preferred Reporting Items for Systematic Reviews and Meta-Analyses (PRISMA) Checklist ^6^ has been included in the *Reporting guidelines* for this review.

### Eligibility criteria

Primary studies had to satisfy four eligibility criteria to be included in the review ( Table 1): two related to the sample of news reports included in the study; one related to the outcome measure; and one related to reporting of the study. We included studies in any language in the qualitative summary, but we only included studies in English or Norwegian in the meta-analyses (the two languages spoken fluently by the primary investigator, MO). Note that the qualitative summary is not a synthesis of qualitative studies, but a structured synthesis of results excluded from the meta-analyses. We did not place any explicit limitation on study design, but—in effect—case studies of a single news report and purely qualitative studies were ineligible.

**Table 1.** Eligibility criteria for primary studies.

**Sample**	*Eligibility Criterion 1:*	Includes content labelled by the researchers as “news”, taken from newspapers or magazines; television, radio or podcasts; or dedicated news websites
	*Eligibility Criterion 2:*	Includes news reports about the effects of health interventions
**Outcome measure**	*Eligibility Criterion 3:*	Includes at least one explicit, prespecified and generic criterion for quality
**Reporting**	*Eligibility Criterion 4:*	Specifies the sampling frame, and selection criteria and technique

The first eligibility criterion was that the sample include content labelled by the researchers as “news”, taken from newspapers or magazines (print); television, radio or podcasts (broadcast); or dedicated news websites (online). We placed no limits on where in the world or when the news reports were published.

Second, the sample had to include news reports about the effects of health interventions. Other types of health news include non-reports (e.g. features or opinion pieces) and other categories of news reports about health (e.g. reports about the health effects of exposures or about health-related ethical or legal issues). We defined “health intervention” as any action intended to improve the health of individuals or communities, as per the Glossary of Evaluation Terms for Informed Treatment choices ( GET-IT). ^7^ We defined “effect” as a negative or positive change or difference in a health outcome, again as per GET-IT.

Third, the researchers had to have used at least one explicit, prespecified and generic criterion to measure quality, herein called a quality criterion. “Quality” was defined as how conducive the report is to informed decisions or, conversely, how misleading it is. Information about the effects of health interventions can be directly misleading—such as claims that an intervention causes an outcome when it is only associated with it—or it can be misleading by omission, such as omitting absolute effect estimates, particularly when the baseline risk is small.

“Criterion” was defined as a standard of quality that could be satisfied or not. “Generic” meant that the quality criterion was not specific to an intervention (e.g. a drug), category of interventions (e.g. “modern” medicine), or condition (e.g. cancer), or specific to any evidence (e.g. findings from a trial). We excluded studies if they only measured the quality of the evidence cited in the news reports; only measured the factual accuracy of the reports; or only described the reports, as opposed to explicitly assessing their quality. We included quality criteria related to cost of the intervention.

The fourth and final eligibility criterion was that the study specified the sampling frame (where the news reports were sampled from), the selection criteria for the news reports, and the selection technique (how the reports were sampled).

To clarify the eligibility criteria, three reviewers piloted them on a sample of five studies from a list of potentially eligible studies of which we were aware before finalising the protocol (Appendix 1 of the protocol; extended data - S2 File ^105^). ^5^

### Search strategy

We searched for eligible primary studies indexed in PubMed on May 24, 2018, and Google Scholar on June 21 and 22, 2018. We searched directly in Open Grey and Grey Literature Report on June 22, 2018, and in ProQuest Dissertations & Theses (Global Full text plus UK and Ireland abstracts) on June 25, 2018. We updated the PubMed search on Aug. 30, 2019.

We conducted citation searches for papers describing the development of tools for assessing the quality of news reports about health: the Index of Scientific Quality; ^8^ the Quality Index for health-related Media Reports; ^9^ and a checklist for improving drug information in the general press. ^10^ We also checked the reference lists of those three papers. Finally, we conducted citation searches and reference lists checks for an arbitrary selection of eligible studies. S5 File in the extended data ^105^ is a detailed description of the search strategy, including search strings.

### Study selection

Screening search results for inclusion in the qualitative summary consisted of two rounds. First, two reviewers screened titles and abstracts. Second, two reviewers screened full texts of studies included after the first round. The two discussed and resolved disagreements after each round. Where two reviewers could not reach a consensus, a third was brought in to arbitrate. Screening reference lists and the results of citation searches included a third, initial round, wherein only one reviewer screened titles. We also screened all of the potentially eligible studies of which we were aware before finalising the protocol (Appendix 1 of the protocol; extended data – S2 file ^105^). We screened search results using Rayyan. ^11^

If a study included a mix of news reports about the effects of health interventions and other types of health news, we excluded the study from the meta-analyses unless results for the news report about the effects of health interventions were reported separately for at least one eligible quality criterion. Further, we excluded studies from the meta-analyses if they did not include any eligible quality criteria or any such criteria for which results were adequality reported (see the section “Assessing criteria”). Judgements about excluding studies from the meta-analyses were made independently by two reviewers, who then discussed and resolved disagreements.

### Data extraction

Data were extracted by a single reviewer and entered into spreadsheets (Microsoft Excel, Version 16.49). We only extracted reported data. For example, if a study did not include the country or countries in which the sample of news reports were published, we did not attempt to code the individual news reports by country or contact the study authors for that information. A second reviewer checked a sample of the extracted descriptive data and checked all the extracted quality criteria that were included in the meta-analyses, as well as results for those criteria.

For the qualitative summary, we extracted descriptive data about the samples of news reports and the tools (sets of quality criteria) used to assess the reports. Data about the sample of news reports included: the categories of media in which the reports were published; the countries in which they were published; the time period in which they were published; the categories of health interventions that were the subject of the reports; and the financial models of the news outlets that published the reports. We also extracted study objectives, sampling frame, selection criteria and technique, and reported subgroup analyses.

All categories and the time periods were predetermined in the protocol. The categories of media were newspaper; magazine; radio; podcast; television; and news website. The categories of intervention were: “modern” medicine; “alternative” medicine; screening; surgery; devices; diet; exercise; lifestyle; and systems and policies. The two categories of financial model were non-commercial (public or independent) and commercial.

We extracted quality criteria verbatim. For the meta-analyses, we extracted all response options for each included quality criterion, the number of relevant news reports (reports about the effects of health intervention) to which the quality criterion was applied, and the results for those reports. Some quality criteria were not applied by researchers to the total sample of relevant reports in a given study, meaning the researchers did not consider the quality criterion universally applicable.

Again, if a study included a mix of news reports about the effects of health interventions and other types of health news, we only included results for the former. We excluded studies if those results were not reported separately.

For the meta-analyses, we were interested in the proportion of relevant news reports that satisfied a given quality criterion. If this was not reported explicitly, we imputed the proportion using reported data, if possible. We excluded quality criteria if the proportion was not explicitly reported and it was impossible to calculate the proportion based on the reported data—for example, if only mean scores were reported.

### Assessing criteria

**Figure 1.**
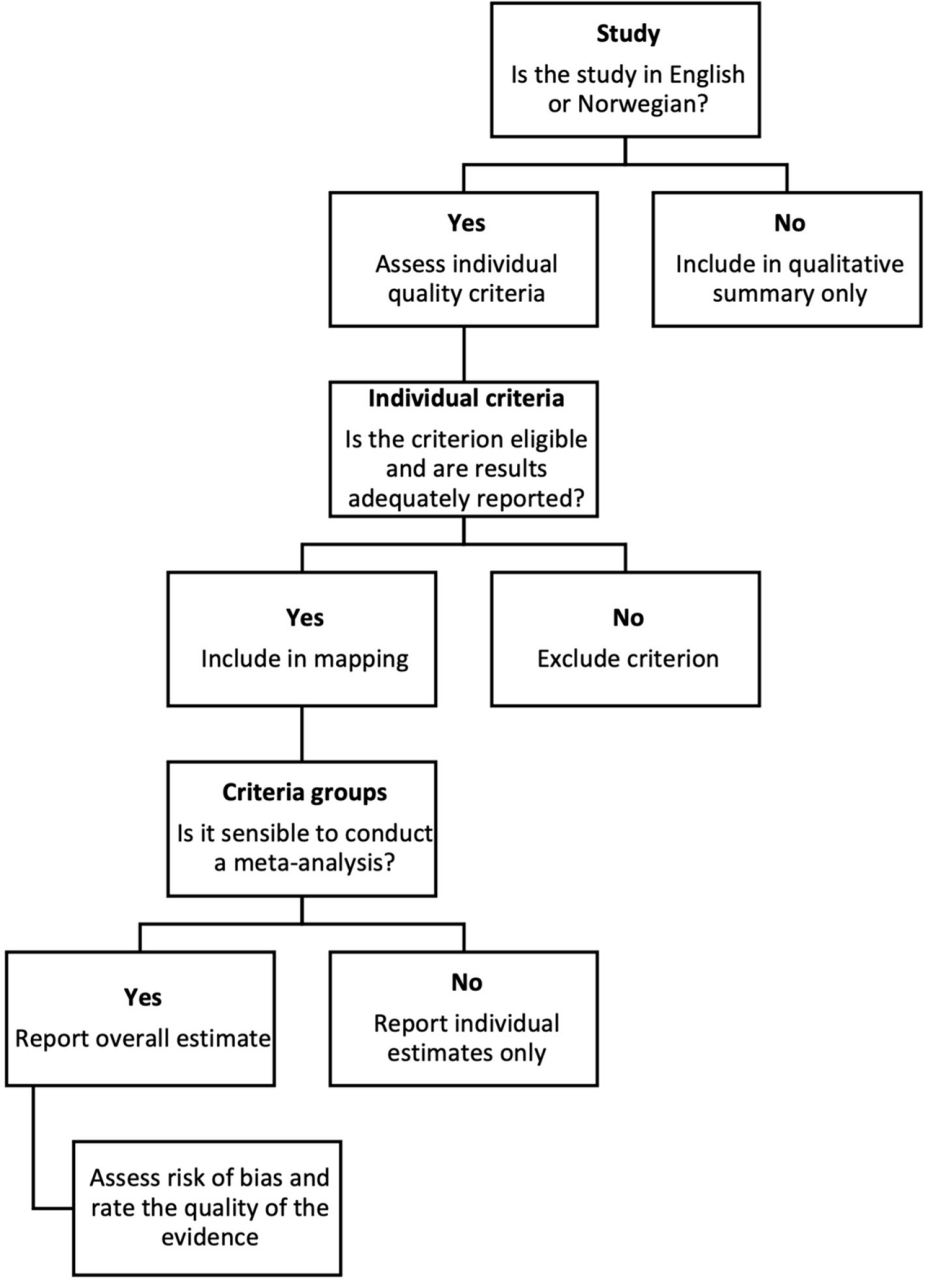
Post-study selection process.

After selecting studies to be included in the meta-analyses ( Figure 1), two authors assessed criteria used in those studies: both quality criteria and other criteria, such as descriptive criteria or criteria measuring factual accuracy, using spreadsheets. We excluded ineligible criteria, as well as criteria for which it was not possible to extract results for only news reports about the effects of health interventions.

We assessed each criterion in each study, even if the researchers used a tool used in a previous study, in case it had been modified. For example, the Media Doctor Australia tool is used in several studies, including those by Smith *et al*. ^12^ and Bonevski *et al*. ^13^ However, while Smith *et al*. asked whether there is “objective evidence to support the treatment”, Bonevski *et al*. asked whether the study methodology is reported, which is more specific.

Two reviewers independently assessed criteria, then discussed and resolved any differences. We excluded criteria if they were non-generic or if they assessed factual accuracy, if they were descriptive, or if they were not clear or sensible (i.e. if their face validity was inadequate ^14^). We also excluded global scores and composite quality criteria because they can be difficult to interpret, and they are difficult to compare across studies using different scoring systems.

### Mapping and grouping quality criteria

We mapped and grouped quality criteria, using spreadsheets, to be able to synthesise results across studies using different tools. For the mapping, we used the IHC Key Concepts framework. The most recent version of the framework was published on IHC website in 2019 and includes nine high-level concepts and 49 low-level concepts organised in three overarching groups (see extended data - S6 File ^105^). ^15^ The IHC Key Concepts are essential concepts for assessing information about the effects of health interventions and making informed choices. ^15– 17^ The framework is based on a mix of evidence and logic. It has a combination of characteristics that make it unique, as well as uniquely useful for this study. First, it has been developed systematically, transparently and iteratively. Second, it only includes concepts that are intended to be relevant to the general public (typically the target audience of health news). And third, it is intended for mapping and assessing measurement tools (amongst other purposes). Moreover, the starting point for the framework included checklists for journalists and input from journalists. ^18^

Two reviewers independently mapped the included quality criteria against the low-level IHC Key Concepts, then discussed and resolved any disagreements. Finally, one reviewer grouped the criteria, informed by the results of the mapping, and a second reviewer assessed whether the groups were sensible (i.e. face validity ^14^).

### Meta-analysis

Where an included quality criterion had more than two response options, one reviewer dichotomised the categories and a second reviewer assessed the dichotomisation. For example, for studies that used the Index of Scientific Quality, where response options range from a score of one to five, we considered a score of four or five as satisfying the quality criterion.

Furthermore, we reframed negative quality criteria as positive, and paraphrased all quality criteria, for the data to be consistently framed and worded, and for the criteria to be succinctly labelled. For example, Bubela *et al*. report the number of news reports that “did not” specify whether a study was randomized (503 of 553, 91%), which was reframed as the number of news reports that did specify whether a study was randomized (50 of 553, 9%). ^19^

For each individual quality criterion and group of criteria, we estimated mean proportions with 95% confidence intervals (CIs), using a random effects model, quantifying precision using 95% confidence intervals. Where criteria groups included more than one criterion from the same study, we included results for both criteria. We performed statistical analysis using Stata 16 (StataCorp LLC, College Station, Texas, USA). Each of the included studies provided data on the number of relevant news reports assessed and how many of those reports met a given criterion. For each study and quality criterion for which necessary data were available, we estimated the proportion of relevant news reports meeting the criterion. These point estimates and 95% confidence intervals were transformed to the logit scale for meta-analysis (Stata's default scale for meta-analysis of proportions). We had anticipated substantial between-study heterogeneity and therefore used random effects meta-analyses. We estimated the percentage of variance that can be attributed to between-study heterogeneity rather than chance (I ^2^, expressed as a percentage) and calculated P-values to test the null hypotheses of no between-study heterogeneity using Cochran's Q statistic. We back-transformed meta-analytical estimates from the logit scale to present estimates as percentages (in forest plots summarizing meta-analytical results from different criteria groups) or proportions (in forest plots for individual groups).

Two reviewers assessed whether what the different quality criteria in each group measured was similar enough that it was sensible to report an overall estimate. If a group included a single quality criterion, we did not report an overall estimate.

### Risk of bias

Two reviewers independently assessed the risk of detection bias for each criterion included in the meta-analyses, then compared judgements and resolved disagreements. Detection bias was assessed using three questions:

1. Was the criterion applied by at least two researchers independently?
2. Were the researchers blinded in terms of the journalist and news outlet?
3. Does the criterion require substantial judgement?

As explained in the protocol (extended data – S2 file), we decided to focus on detection and selection bias based on the critical appraisal tool developed by Munn *et al*. for systematic reviews of observational epidemiological studies reporting prevalence data, ^20^ the Strengthening the Reporting of Observational Studies in Epidemiology (STROBE) statement, ^21^ and methods used by the Cochrane Methodology Review Group to systematically review the prevalence of an outcome in a population of information (as opposed to people). ^22^ As explained in this article, in the section on deviations from the protocol, we later found that the item we had developed to assess selection bias told us more about applicability than bias, and therefore only assessed detection bias.

If the authors did not state how many researchers applied the criterion or whether the researcher(s) did so independently, we recorded the answer to the first question as “unclear” and counted it as a “no”. If from looking at the criterion in and of itself, it was unclear how much judgement was required, we looked at whether the researchers reported or referenced any guidance for applying it. If they did, we looked at how detailed and specific the guidance was. If the researcher(s) applying the criterion received training, but the authors did not report what this entailed—i.e. what guidance was provided—we considered the criterion to require substantial judgement.

We considered the risk of detection bias as low if the criterion did not require substantial judgement. We considered the risk of detection bias as moderate if the criterion required substantial judgement, but at least two researchers applied the criterion independently and they were blinded in terms of the journalist and outlet. We considered the risk of detection bias as high if the criterion required substantial judgement and only one researcher applied it or the researchers were not blinded.

If there was a high risk of bias for criteria providing ≥50% of the weight in a given criterion group, we considered there to a be a high risk of bias for the overall estimate.

### Rating the quality of the evidence

We used the Grading of Recommendations Assessment, Development and Evaluation (GRADE) approach as a framework for rating the quality of evidence. ^23^ GRADE considers five factors that can lower the certainty of evidence for effect estimates: risk of bias, inconsistency of results, indirectness of evidence, imprecision, and publication bias. We did not consider publication bias, as we are not aware of any research documenting publication bias in studies of the quality of news reports or similar studies. We also did not consider directness, which was irrelevant.

Again, if there was a high risk of bias for criteria providing ≥50% of the weight in a given group, we considered there to be a high risk of bias for the overall estimate. If a CI is wider than 0.25 (a quartile), we considered there to be important imprecision, based on guidance from the Cochrane Effective Practice and Organization of Care (EPOC) group. ^24^ We used an I ^2^ of >75% as a rule of thumb for there being substantial inconsistency.

GRADE factors for increasing the certainty of the evidence were irrelevant.

### Deviations from the protocol

We report the main deviations from the protocol here. In S7 File (see extended data ^105^), we provide detailed information about all deviations. Some of the deviations were in part due to the originality of our research question in terms of a systematic review and meta-analysis, meaning there was lack of methodological guidance or precedence for the study as a whole. In other words, we were in “unchartered waters”. Other deviations were due to limited time and resources. This review had no funding and as noted in the background section, the first part of the review was MO's MSc dissertation, meaning he was required to do as much as possible of that work himself.

In terms of search strategy, we did not search Scopus as planned, aside from citation searches, since the time saved outweighed the likelihood of identifying additional eligible studies, based on the results of searching the other databases. Also, we only conducted reference list checks and citation searches for arbitrary samples of included studies, due to limited time and resources, as well as decreasing yield (extended data – S5 File ^105^).

In terms of eligibility, we made explicit that at least one of the criteria that researchers in a given study used to assess quality had to be prespecified and generic, and used specifically to assess the quality of news reports, not describe their content or assess the quality of underlying evidence. As mentioned, we included studies in any language in the qualitative summary, but only included studies in English and Norwegian (the two languages spoken fluently by the primary investigator, MO) in the meta-analyses.

In terms of data extraction, a second reviewer checked all data extracted for the meta-analyses, but only a sample of data extracted for the qualitative summary due to limited time and resources. There were no errors in the sample. In terms of assessing risk of bias, we were originally going to assess selection bias by looking at how news reports were sampled: randomly, sequentially or other. However, we found this told us more about applicability than bias, since samples typically consisted of all news reports that met the researchers’ eligibility criteria, rather than a sample of those reports. Therefore, we only assessed the risk of detection bias, as described earlier.

In terms of synthesising results and rating the quality of the evidence, we have not reported overall estimates for groups of quality criteria if what those criteria measure is too different for a synthesis to be sensible, despite the criteria being clearly related. Nor have we reported overall estimates for groups that include a single criterion. Further, we did not rate the quality of the evidence in these cases. Where we do report an overall estimate and rate the certainty of the evidence, if we assessed the risk of bias as high for criteria providing ≥50% of the weight in a given group, we assessed the risk of bias as high for the group as a whole. We did not consider assessing risk of bias for whole criteria groups in the protocol.

## Results

**Figure 2.**
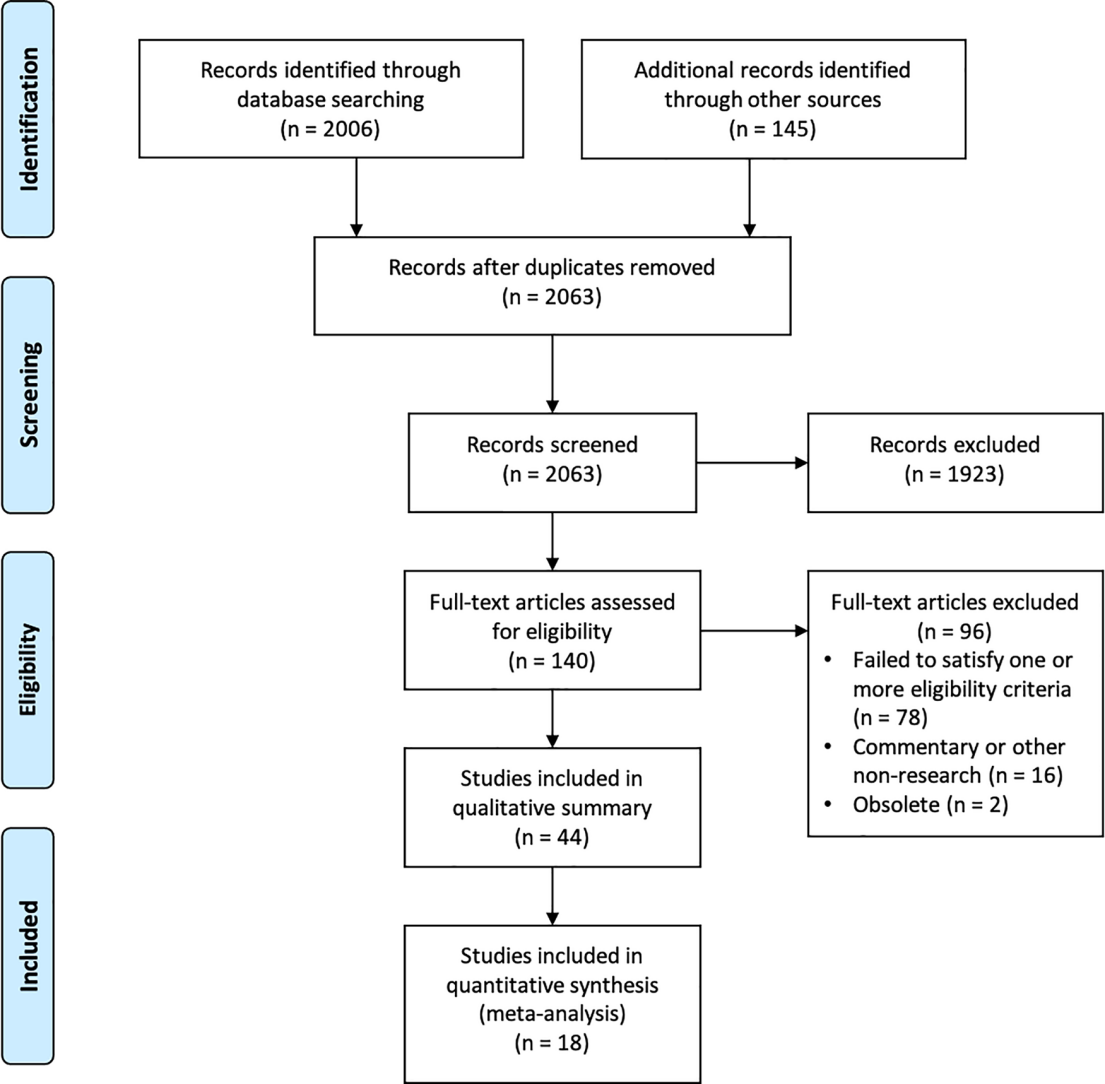
PRISMA flow diagram.

We screened 2063 unique records and retrieved and assessed 140 full texts for eligibility, as shown in the PRISMA flow diagram ( Figure 2). We identified 44 primary studies for inclusion in the qualitative summary (see extended data - S1 Table ^105^): 31 in English, five in German, four in Spanish, three in Norwegian and one in Slovenian. We excluded most (78) full-text articles from the qualitative summary based on one or more of the four eligibility criteria. We excluded another 16 full-text articles that were not research (e.g. commentaries), and two duplicates: one doctoral thesis that is a composite of other studies that we assessed ^25^ and one working paper ^26^ superseded by the final publication. ^27^ The third eligibility criterion—that the researcher had used at least one explicit, prespecified and generic quality criterion—was least often satisfied. We excluded 47 full-text articles solely for failing to satisfy that eligibility criterion.

Of the 44 studies included in the qualitative summary, we included 18 (41%) in the meta-analyses. Of the 26 studies only included in the qualitative summary, we excluded nine (20%) from the meta-analyses for being in a language other than English or Norwegian; 16 for inadequate reporting (36%); and one (2%) where the sample of news reports ^28^ was included in a larger sample in a later study. ^29^ Only one of the 18 studies included in the meta-analyses was in Norwegian, ^30^ meaning the rest were in English.

### Study characteristics

**Samples**

Table 2 is an overview of the samples of news reports in the studies included in the qualitative summary. Descriptive data for each study can be found in S2 Table in the extended data.

**Table 2.** Characteristics of samples included in the qualitative summary.

Medium ^a^	Studies ^b^	Country	Studies ^b^	Time period ^c^	Studies ^**b**^	Intervention category ^d^	Studies ^**b**^
Magazines	10	Argentina	1	1990-1999	7	“Alternative” medicine	4
News websites	8	Australia	5	2000-2009	23	Devices	3
Newspapers	35	Canada	5	2010-2019	19	Diet	7
Other (Print) ^e^	1	Germany	5			Exercise	4
Podcasts	0	Italy	2			Lifestyle	4
Radio	1	New Zealand	2			“Modern” medicine	24
TV	7	Norway	2			Other	7
Unspecified	5	Slovenia	1			Screening	5
		South Africa	1			Surgery	6
		Spain	3			Systems and policies	3
		Trinidad and Tobago	1			Unspecified	12
		United Kingdom	7				
		United States	12				
		Vietnam	1				
		Unspecified	4				

^a^ As per categories pre-specified in the protocol: newspapers, magazines, TV, radio, podcasts, and news websites.

^b^ The same study may be counted in several categories.

^c^ Time period when news reports were published or studied, depending on what authors reported.

^d^ As per categories pre-specified in the protocol: “modern” medicine, “alternative” medicine, screening, surgery, devices, diet, exercise, lifestyle, and systems and policies.

^e^ The authors do not specify whether the news reports were sampled from newspapers, magazines or both.

Sample sizes varied from 17 to 3096 units. We use “units” here since some samples include content besides news reports, such as non-news websites, ^31^ or include health news that is not news reports about the effects of health interventions, for example news features ^32^ or news reports about the effects of exposures, such as recreational cannabis (as opposed to medical cannabis). ^33^

Newspaper was the most common medium, with newspaper content explicitly included in 35 of the 39 studies (90%) in which a medium was specified ( Table 2). However, the number of studies that included newspaper content versus content from news websites should be interpreted with caution, since newspaper content may also have been published online. In the study with the second-largest sample, Walsh-Childers *et al*. write that news reports “must have been published on the news organization's website,” but categorise those reports only as newspaper content. ^29^

The United States (US) was the most common country in terms of where content assessed in the included studies was published. Of the 40 studies where at least one country was specified, 12 samples (30%) included content published in the US ( Table 2). Twenty-five (63%) of those 40 studies included content published in English-speaking countries (see extended data - S2 Table ^105^).

We categorised time periods according to what authors reported. This was in some cases the period in which news reports were published and in others the period in which reports were assessed. Differences between the two are likely minor, since all studies focused on the recent past (relative to the time that the study was conducted). The earliest year of publication was 1996. ^34^

“Modern” medicine was included in 24 of 32 samples (75%) where a category of interventions is specified. In some cases, it was not possible to use the categories pre-specified in the protocol for this review. For example, Haneef *et al*. included news reports about “non-pharmacological” interventions, ^35^ which could include “Alternative” medicine, devices, diet, etc. In these cases, we used the category “Other”.

Financial model was not specified in any of the included studies—i.e. it was not explicitly reported whether the news outlets that published news reports included in the sample were commercial or non-commercial (public or independent).

**Tools**

The tools used to assess the quality of news reports in each study are presented with the descriptive data in S2 Table in the extended data. ^105^ Most studies used a tool that was, to varying degrees, original or modified. Only one tool was used in >4 of the 44 studies included in the qualitative summary: the Index of Scientific Quality, which was used in seven studies (16%). ^31, 33, 34, 36– 39^ However, even more studies used tools that were to some extent, directly or indirectly, based on the criteria developed by Moynihan *et al*. ^40^ This includes the four studies that used the Media Doctor Australia tool ^12, 13, 41, 42^ and the 3 studies that used the Health News Review tool, ^28, 29, 43^ which was based on the Media Doctor Australia tool. ^28^ Two studies used two separate tools. ^41, 44^ We did not identify any studies that used the Quality Index for health-related Media Reports ^9^ or the checklist for improving drug information in the general press. ^10^

**Objectives, sampling frames, selection criteria and technique, and reported subgroup analyses**

The explicit or implicit objectives of studies included in the qualitative summary are presented in S3 Table, the sampling frames and selection criteria and techniques in S4 Table, and the variables used in the reported subgroup analyses in S5 Table (see extended data ^105^). Detail and clarity varied. For example, some studies reported explicit inclusion and exclusion criteria for news reports, while others simply stated that they assessed news reports that included health or medical information.

Thirty-one studies (70%) reported at least one subgroup analysis, exploring differences in quality between subgroups of news reports. The most common overarching variables were ones related to time, medium, and outlet. How subgroups were categorised for the same overarching variable, as well as what categories were included, varied. For example, some studies included subgroup analyses based on specific intervention (e.g., comparing the quality news reports about one medicine with news reports about another medicine). Others included subgroup analyses based on the category of intervention (e.g., comparing news reports about “modern” medicines versus news reports about other categories of interventions). Furthermore, the specific interventions or categories of interventions varied.

### Assessment, mapping and grouping of criteria

Two reviewers assessed all 208 criteria applied to news reports in the 18 studies included in the meta-analyses. Not all criteria were measures of quality. Of the 208 criteria, 77 (37%) were excluded: 66 (32%) for being non-generic or descriptive, for measuring factual accuracy, or for not being clear or sensible (having inadequate face validity); and 11 (5%) for being global scores or composites. We provide examples in Table 3. Quality criteria were judged not to be sensible if they were in conflict with the IHC Key Concept and research evidence, for example a criterion assessing whether research presented in a news report is peer-reviewed. ^43, 45^ The same two reviewers mapped the remaining 131 of 208 quality criteria (63%) against the IHC Key Concepts. Based on the results of the mapping, we established 19 groups of quality criteria (criteria groups), as well as eight subgroups.

**Table 3.** Examples of criteria individually excluded from meta-analyses.

Reason	Criterion	Explanation	Citation
*Non-generic*	“Potential risks or harm”; “Accordance with available evidence”	The criterion measures whether risks or harms mentioned in the news report are consistent with specific evidence, not whether risks or harms are mentioned or discussed.	Robinson 2018
*Descriptive*	“Picture/Illustration”	The criterion is a descriptive variable about whether the news report includes a picture or illustration, not intended to be a measure of quality.	Finer 1999
*Measuring factual accuracy*	«Is the treatment genuinely new?»	The criterion is a measure of whether information in the news report is true, as opposed to informative (assuming it is true).	Bonevski 2008
*Unclear*	«Inadequate claim of safety»	There is no explanation of “inadequate”, making it impossible to assess whether the criterion is sensible.	Yavchitz 2012
*Not sensible*	«Was the research peer reviewed?»	Peer review is a poor indicator of quality. ^45^	Stassen 2016
*Global score or composite*	«News with at least one misleading reporting»	This was a composite of several criteria measuring different types of misleading news reporting.	Haneef 2015

In S6 Table in the extended data, ^105^ we present the number of quality criteria included in the meta-analyses per relevant IHC Key Concept. We were unable to identify or include quality criteria related to several IHC Key Concepts that are important for communicating information about the effects of health interventions in the news media or otherwise. For example, there were no criteria related to the concept “ *Average differences between treatments can be misleading*” (2.3c).

### Synthesis of results

Of the 208 criteria used in the 18 studies included in the meta-analysis, we included 108 (52%) in the meta-analyses. We calculated overall estimates for five of the 19 main criteria groups (26%) and 6 of the 8 subgroups (75%). In those cases, we found:

• 86% of news reports do not commit “disease mongering” (95% CI 78%-91%) (low certainty),
• 22% adequately address conflicts of interest (95% CI 7%-49%) (low certainty),
• 93% do not selectively report “statistically significant” results (95% CI 87%-97%) (high certainty),
• 53% quantify effects (95% CI 36%-69%) (low certainty),
• 68% do not use misleading language (95% CI 35%-90%) (low certainty),
• 17% report absolute effects (95% CI 4%-49%) (low certainty),
• 36% include adequate information about alternative interventions (95% CI 26%-47%) (moderate certainty),
• 82% do not focus on surrogate outcomes or extrapolate from surrogate to important outcomes (95% CI 76%-86%) (high certainty),
• 40% mention, discuss or explain potential harms of the intervention (95% CI 23%-61%) (low certainty),
• 18% mention or discuss the cost of the intervention (95% CI 12%-28%) (moderate certainty), and
• 97% mention at least one benefit (95% CI 56%-100%) (moderate certainty).

Of the 131 quality criteria included in the 19 groups, we excluded 22 (17%) from the meta-analyses for inadequate reporting of results, meaning it was impossible to impute the proportion of relevant news reports that satisfied the criterion based on the reported data. The distribution of the remaining 108 criteria amongst the 19 main groups and eight subgroups, is presented in Table 4, with the order of the groups based on the IHC Key Concept framework. In one case, we included a criterion in two groups: the third criterion in the study by Stassen, ^43^ which we included in Criteria related to qualitative descriptions of effects and Criteria related to pros and cons. Several criteria from the same study were included in three of the 19 main groups (16%) and four of the eight subgroups (50%). Criteria related to “disease mongering” ^12^ stem from a paper by Moynihan *et al.* ^46^

**Table 4.** Criteria groups. N = criteria included in the meta-analyses.

Criteria related to	N	Subgroups	N
Associations and randomisation	3		
Need for comparisons	3		
Consistency between studies	1		
“Disease mongering" ^46^	5		
Conflicts of interest	7		
Opinions	1		
Study design and risk of bias in general	5		
Blinding	1		
Loss to follow-up	1		
Selective reporting	2		
Qualitative descriptions of effects ^a^	8	Quantification of effects ^a^	6
		Misleading language	2
Absolute effects	5		
Play of chance	4		
Subgroup analyses	1		
Lack of evidence	1		
Options	7		
Extrapolations ^a^	21	Extrapolation (outcome)	2
		Extrapolation (population) ^a^	8
		Extrapolation (intervention)	8
Animal studies	1		
Pros and cons ^a^	32	Harms ^a^	14
		Cost ^a^	11
		Benefits	2

^a^ Includes multiple criteria from the same study.

**Figure 3.**
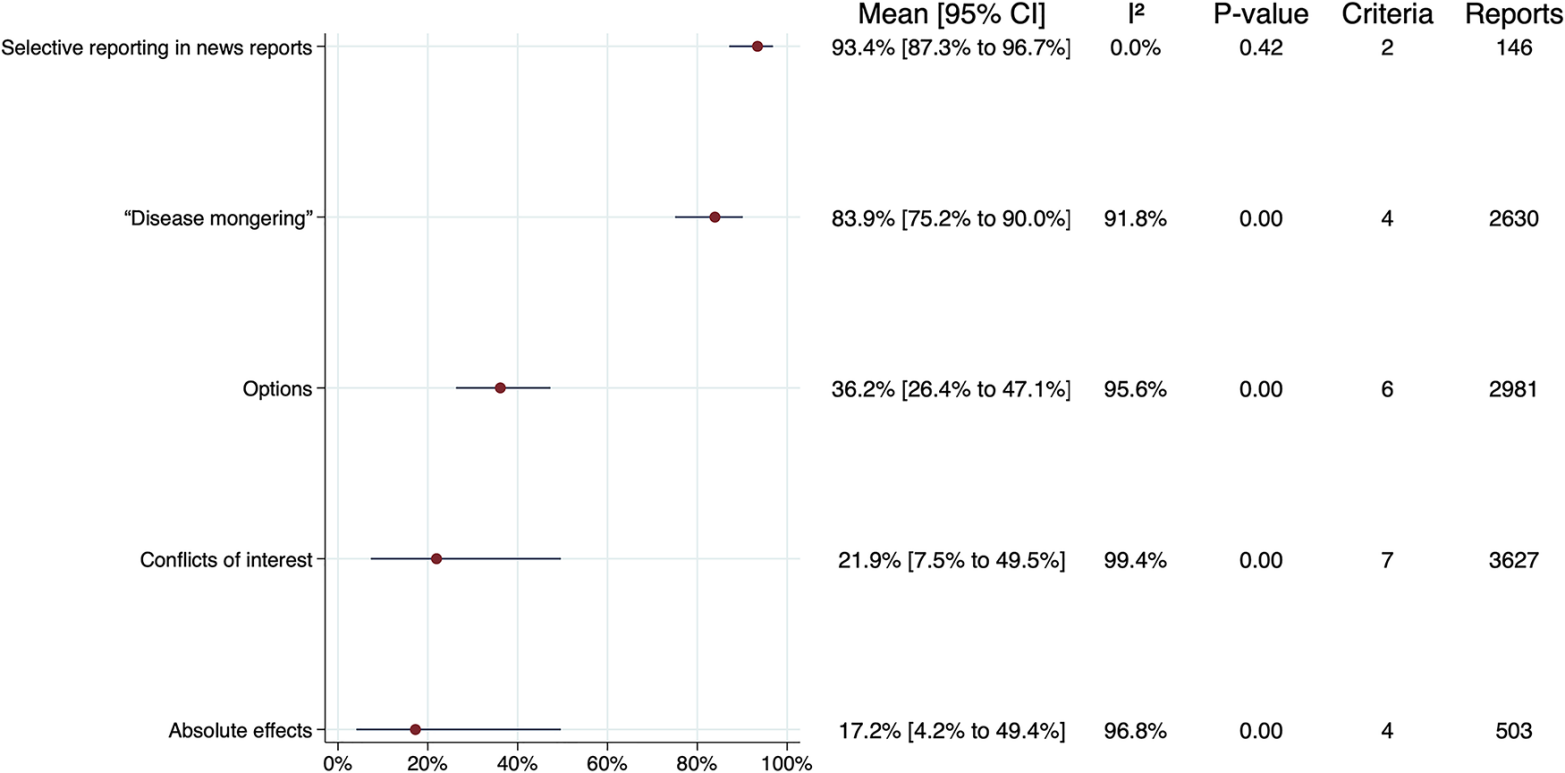
Summary of primary meta-analyses.

**Figure 4.**
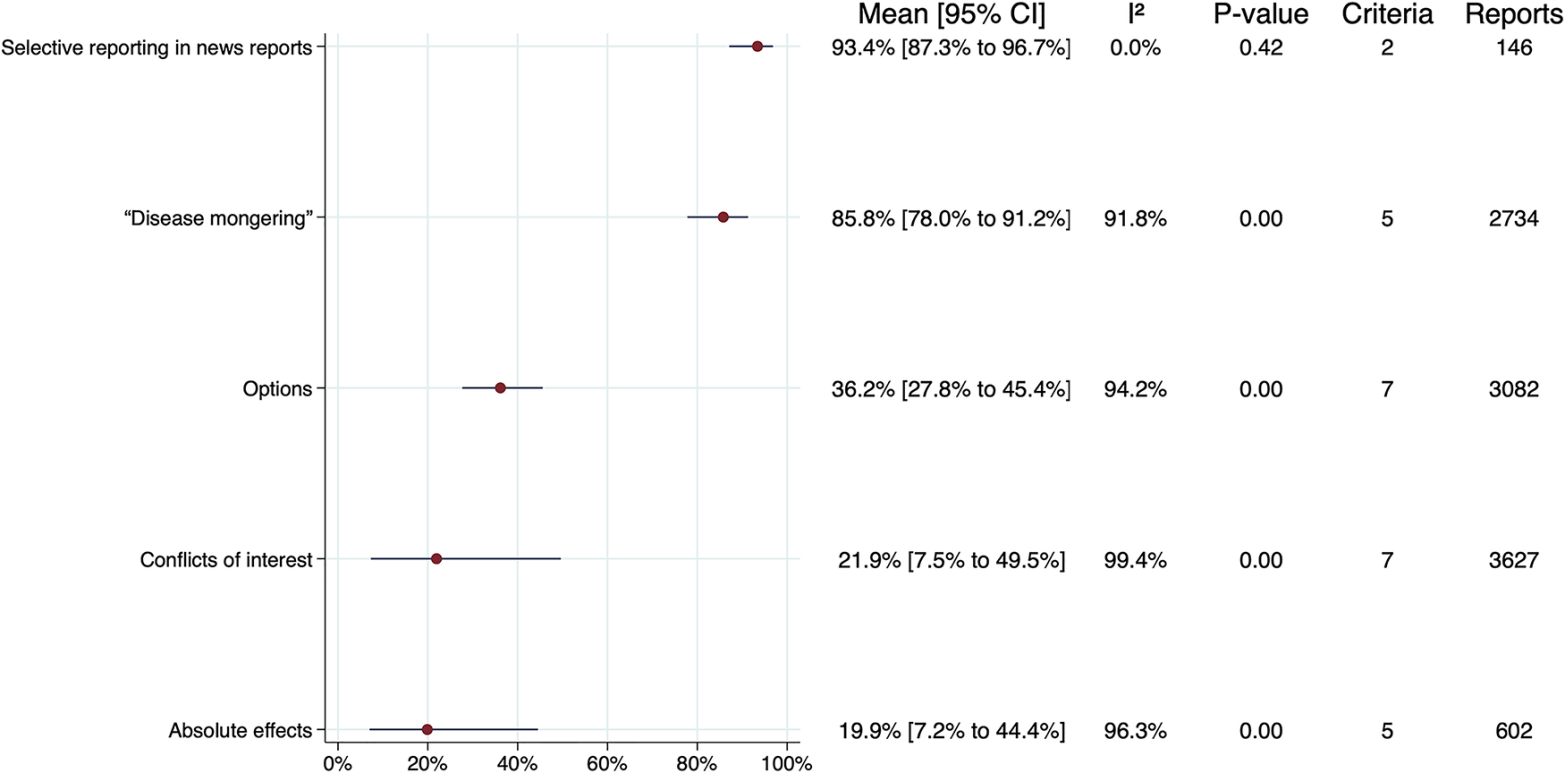
Summary of sensitivity analyses.

The primary meta-analyses are summarised in Figure 3, excluding subgroups. The samples of news reports in two of the studies included in the meta-analyses overlap. ^12, 13^ For groups that included a criterion from both of these studies, we performed primary analyses that included only the largest and most-recent study by Bonevski *et al*., ^13^ and an ad hoc sensitivity analysis that also included results from the study by Smith *et al.* ^12^ The sensitivity analyses are summarised in Figure 4, again excluding subgroups. There were no important differences between the overall estimates and CIs for the primary analyses versus the sensitivity analyses. Forest plots for each criteria group, including individual estimates and CIs for each criterion, are presented in S7 File in the extended data. ^105^

For most of the main groups (14 of 19) and a minority of the subgroups (two of eight), we did not consider it sensible to report overall estimates because—although the criteria in the group are clearly related—what they each measure was too different for it to be sensible to synthesise results. For example, in the group Criteria related to associations and randomization, the ninth criterion from the study by Bubela *et al*. measured whether the news report specifies that a trial was randomized or not, whereas the 12 ^th^ criterion from the study by Haneef *et al*. measures whether there is a causal claim based on a non-randomized study. The two are clearly related, but at the same time, synthesising results would clearly not be sensible. Leaving out whether a study was randomized might be misleading by omission, but this can be compensated for by distinguishing causation and association and pointing out potential confounding. On the other hand, making a causal claim based on a non-randomized study is directly misleading. Further, we do not report overall estimates for the groups that include a single criterion.

Due to heterogeneity, as well as inadequate reporting and small sample sizes in some studies, we did not conduct any of the six subgroup analyses considered in the protocol, exploring differences in quality between news reports:

1. in different media (broadcast vs. other),
2. from different decades,
3. by commercial vs. non-commercial outlets,
4. by health or science journalists vs. others,
5. in low-income countries vs. middle or high-income countries, or
6. in broadsheet vs tabloid newspapers. ^5^

**Risk of bias**

The individual risk of bias assessments for each of the 108 criteria included in the meta-analyses are presented in S9 File in the extended data. ^105^ We considered there to be a high risk of bias in 13 cases (11%), including in the case of the one criterion included in two criteria groups (the third criterion in the study by Stassen ^43^). The 13 criteria for which we considered there to be a high risk of bias are the 13 that required substantial judgement to be applied, according to our assessment. In 12 of those 13 cases, it was—in addition—unclear whether researchers were blinded to the publication and journalist behind the news report. In the remaining case, it was clear that they were not blinded. In four of the 13 cases, it was also unclear whether the criterion was applied independently by at least two researchers.

**Quality of the evidence**

We only assessed the quality of the evidence for the five main groups and six subgroups where we report an overall estimate. Quality varied from low to high. In all but two of 10 cases, we downgraded quality for substantial inconsistency. In half of the cases, we downgraded for important imprecision. Finally, for the group criteria related to “disease mongering”, we downgraded for overall high risk of bias.

### Results by criteria group

We summarise the main findings here. Details of these analyses are reported in S10 File in the extended data. ^105^

**Criteria related to associations and randomization**

Many news reports included causal claims based on associations, and few said whether trials were randomized. We included three estimates, from three studies, ^19, 35, 47^ related to the IHC Key Concepts that *“An outcome may be associated with a treatment but not caused by it”* (1.2d) and *“Comparison groups should be as similar as possible”* (2.1a). Based on estimates for single criteria with a low risk of bias, 51% of news reports make causal claims based on non-randomized studies (95% CI 40%-62%) ^32^ and 9% specify whether a given trial was randomized (95% CI 7%-12%). ^19^

**Criteria related to the need for comparisons**

Some news reports concluded that an intervention has an effect despite a lack of a comparator, and many did not include evidence to support the effectiveness of the intervention. We included three estimates, from three studies, ^12, 35, 41^ related to the IHC key concept that *“Identifying effects of treatments depends on making comparisons”* (1.2f). Based on an estimate from a single study, three quarters (74%) of news reports avoid concluding there is a beneficial effect when there is a lack of a comparator (95% CI 63%-83%). ^35^ Two studies found that only a third (34%) ^12^ to half (55%) ^41^ of news reports include evidence about the effects of the intervention (95% CIs 25%-43% and 38%-72%). The risk of bias for these two criteria was high.

**Criteria related to consistency between studies**

Few news reports considered and soundly assessed the consistency of evidence. We included a single criterion, from the study by Krauth and Apollonio, ^31^ related to the IHC Key Concept that *“The results of one study considered in isolation can be misleading”* (1.2g) and *“Consider how certain you can be about each advantage and disadvantage”* (3.3d). The risk of bias was low for this criterion. The study found that 13% (95% CI 7%-26%) of news reports published in American newspapers and magazines about tobacco cessation therapy “consider” and make a “well-founded” assessment of consistency between studies.

**Criteria related to “disease mongering”**

Some news reports committed disease mongering. We included five estimates, from five studies, ^12, 13, 29, 41, 43^ related to disease mongering, which is addressed by the IHC Key Concept that *“Earlier detection of ‘disease’ is not necessarily better”* (1.2k). All five criteria had a high risk of bias. Overall, 86% of news reports do not commit disease mongering (95% CI 78%-91%) ( Figure 3). The certainty of the evidence for that estimate is low due to substantial inconsistency (I ^2^ = 92%) and the high risk of bias.

**Criteria related to conflicts of interest**

Many news reports failed to address conflicts of interest. We included seven estimates, from seven studies, ^19, 29, 30, 40, 41, 43, 48^ related to the IHC Key Concept that *“Competing interests may result in misleading claims”* (1.3b) All criteria had a low risk of bias. Overall, 22% (95% CI 7%-49%) in some way disclose potential conflicts of interest ( Figure 3). The certainty of the evidence for that estimate is low, due to substantial inconsistency (I ^2^ = 99%) and important imprecision.

**Criteria related to opinions**

Some news reports failed to adequately distinguish opinion and fact. We included a single estimate, from the study by Krauth and Apollonio, ^31^ related to the IHC Key Concept that *“Opinions alone are not a reliable basis for claims”* (1.3d). The study evaluated American newspaper and magazine reports about tobacco cessation therapy. It found that three quarters (73%) of news reports adequately distinguish opinion and fact (95% CI 6%-83%). The criterion had a low risk of bias.

**Criteria related to study design and risk of bias in general**

Many news reports did not address the “quality of evidence”. We included five estimates, from five studies, ^13, 29, 31, 41, 43^ related to the risk of bias (“type”, “credibility” or “quality” of the evidence), which is addressed by multiple low-level IHC Key Concepts under the high-level concepts *“Comparisons of treatments should be fair”* (2.1) and *“Syntheses of studies need to be reliable”* (2.2). Three of the criteria had a high risk of bias. The proportion of the news reports that meet the criterion used in each study varied from 8% (95% CI 3%-20%) ^31^ to 77% (95% CI 73%-81%). ^43^

**Criteria related to blinding**

Few news reports said whether trials were “double blinded”. We included a single criterion, from the study by Bubela *et al*., ^19^ related to the IHC Key Concepts that *“The people being compared should be cared for similarly apart from the treatments being studied”* (2.1c), *“If possible, people should not know which of the treatments being compared they are receiving”* (2.1d) and *“Outcomes should be assessed in the same way in all the groups being compared”* (2.1e). The study evaluated reports in newspapers in English-speaking countries about herbal remedy and conventional pharmaceutical trials. It found that 7% (95% CI 6%-9%) of news reports specify if a trial was “double-blinded”. The criterion had a low risk of bias. The term “double blind” is a problematic term since it can have several meanings. ^40^ The study did not address this.

**Criteria related to loss to follow-up**

Few news reports said anything about follow-up of participants in trials. We included a single criterion, from the study by Bubela *et al*., ^19^ related to the IHC Key Concept that *“It is important to assess outcomes in all (or nearly all) the people in a study”* (2.1g). This was the same study that evaluated whether news reports specify whether a trial was “double blind”. ^18^ The risk of bias was low for this criterion. The study found that 7% (95% CI 5%-9%) of news reports specify withdrawals or dropouts from a trial.

**Criteria related to selective reporting**

Few news reports selectively reported “statistically significant” results. We included two estimates, from two studies, ^35, 47^ related to IHC Key Concepts that *“Failure to consider unpublished results of fair comparisons may result in estimates of effects that are misleading”* (2.2b) and *“Deeming results to be ‘statistically significant’ or ‘nonsignificant’ can be misleading”* (2.3g). Overall, 93% (95% CI 87%-97%) of news reports avoid selectively reporting “statistically significant” results ( Figure 3). The certainty of the evidence for that estimate is high.

**Criteria related to qualitative descriptions of effects**

Many news reports used misleading language to describe intervention effects and did not quantify effects. We included seven estimates, from five studies, ^29, 30, 35, 40, 43^ related to the IHC Key Concept that *“Verbal descriptions of the size of effects alone can be misleading*” (2.3a) Overall, four studies that reported six criteria related to quantification of effects found that 53% (95% CI 36%-69%) of news reports quantify effects, as opposed to only describing them qualitatively. There was a high risk of bias for two of the criteria. The certainty of the evidence for this estimate is also low, due to substantial inconsistency (I ^2^ = 99%) and important imprecision. Overall, two studies, found that 68% (95% CI 35%-90% of news reports do not use misleading language to describe intervention effects. One of the criteria had a risk of bias. The certainty of the evidence for that estimate is low, due to substantial inconsistency (I ^2^ = 98%) and important imprecision.

**Criteria related to absolute effects**

Most news reports did not report absolute effects. We included five estimates, from five studies, ^12, 13, 40, 41, 49^ related to the IHC Key Concept that *“Relative effects of treatments alone can be misleading”* (2.3b). All five criteria had a low risk of bias. Overall, 17% (95% CI 4%-49%) of news reports report absolute effects ( Figure 3). The certainty of the evidence for that estimate is low, due to substantial inconsistency (I ^2^ = 97%) and important imprecision.

**Criteria related to the play of chance**

Few news reports claimed that an intervention has a benefit despite a small sample size, and more than half specified the sample size of trials, but few clearly and soundly “assessed the precision” ^8^ of reported estimates of effect. We included four estimates, from four studies, ^19, 31, 35, 39^ related to the IHC Key Concepts that *“Small studies may be misleading”* (2.3d) and *“The use of p-values may be misleading; confidence intervals are more informative”* (2.3f). Based on estimates from single studies 95% (95% CI 88%-98%) of news reports do not claim there was a benefit despite a small sample size, ^35^ and 63% (95% CI 59%-67%) specify the sample size of trials. ^19^ Two studies found that 6% (95% CI 1%-38%) ^39^ and 8% (95% CI 3%-20%) ^31^ of news reports “clearly and soundly” assess the precision of estimates or risk of random error.

**Criteria related to subgroup analyses**

Most news reports did not focus on inappropriate subgroups. We included a single estimate, from the study by Yavchitz *et al*., ^47^ related to the IHC Key Concept that *“Results for a selected group of people within a study can be misleading”* (2.3e). The study found that 94% (95% CI 85%-98%) of news reports avoid focusing on an “inappropriate subgroup”. The criterion had a low risk of bias.

**Criteria related to lack of evidence**

Most news reports avoided claiming that interventions have equivalent effects based on a difference that was not “statistically significant”. We included a single estimate, from the study by Yavchitz *et al*., ^47^ related to the IHC Key Concept that *“Lack of evidence of a difference is not the same as evidence of ‘no difference’”* (2.3h). The study found that 93% (95% CI 83%-97%) of news reports do not claim equivalence when there was a “statistically nonsignificant” difference. ^47^ The criterion had a low risk of bias.

**Criteria related to options**

Most news reports did not include information about alternative interventions. We included seven estimates, from six studies, ^13, 29, 41, 43, 50^ related to the IHC Key Concept *“Be clear about what the problem or goal is and what the options are”* (3.1a). Overall, 36% (95% CI 26%-47%) of news reports include information about alternative interventions ( Figure 3). The certainty of the evidence for that estimate is moderate, due to substantial inconsistency (I ^2^ = 96%).

**Criteria related to extrapolations**

Some news reports focused on surrogate outcomes or extrapolated from surrogate to important outcomes. Some news reports did not report indications for interventions and most did not report contraindications. Few news reports did not clearly report to whom the information applied. Some extrapolated from study participants to a larger or different population. Many news reports did not consider the availability of the intervention or the dosage of the intervention. Some interventions extrapolated from the study intervention to a different intervention.

Altogether, we included 21 criteria, from 12 studies, ^12, 13, 19, 29, 31, 35, 38, 41, 43, 47, 50, 51^ related to the IHC Key Concepts that *“Attention should focus on all important effects of treatments, and not surrogate outcomes”* (3.2a), *“Fair comparisons of treatments in animals or highly selected groups of people may not be relevant”* (3.2b), *“The treatments compared should be similar to those of interest”* (3.2c) and *“There should not be important differences between the circumstances in which the treatments were compared and those of interest”* (3.2d). There was a low risk of bias for all the criteria.

We included two criteria, from two studies ^35, 50^ related to the IHC Key Concept that *“Attention should focus on all important effects of treatments, and not surrogate outcomes”* (3.2a). Overall, 82% (95% CI 76%-86%) of news reports do not solely focus on surrogate outcomes or extrapolate from surrogate to important outcomes. The certainty of the evidence for this estimate is high.

We included eight criteria, from six studies, ^31, 35, 38, 41, 50, 51^ related to IHC Key Concept that *“Fair comparisons of treatments in animals or highly selected groups of people may not be relevant”* (3.2b). Five of these criteria, from three of the studies, ^41, 50, 51^ addressed reporting of indications and contraindications. Indications are reported in 70% (95% CI 64%-76%) to 97% (95% CI, 95%-98%) of news reports, whereas contraindications are only reported in 4% (95% CI 2%-8%) to 13% (95% CI 11%-16%) of news reports. Two criteria, from two studies, ^31, 38^ addressed whether it was clear to whom the information applies. These criteria are met in 94% (95% CI 81%-98%) to 100% (95% CI 98%-100%) of news reports. The other criterion, from the study by Haneef *et al*., ^35^ assessed whether results were extrapolated from study participants to a larger or different population. The study found that 85% (95% CI 72%-92%) of news reports do not extrapolate to a larger or different population.

We included eight criteria, from eight studies, ^13, 19, 29, 35, 41, 43, 51^ related to the IHC Key Concept that *“The treatments compared should be similar to those of interest”* (3.2c). Five of these criteria addressed availability of the intervention. The availability of the intervention is reported in 41% (95% CI 37%-45%) to 79% (95% CI 66%-88%) of news reports. Two of the criteria addressed dosage. Those studies found that 14% (95% CI 11%-17%) ^19^ to 50% (95% CI 46%-54%) ^51^ of news reports specify or mention dosage. The other criterion addressed extrapolation from the study intervention to a different intervention. That study ^35^ found that 85% (95% CI 72%-92%) of news reports do not extrapolate to a different intervention.

**Criteria related to animal studies**

Some news reports extrapolated a beneficial effect from an animal study to humans. We included a single estimate, from the study by Haneef *et al*., ^35^ related to the IHC Key Concept that *“Fair comparisons of treatments in animals or highly selected groups of people may not be relevant”* (3.2b). The study, which had a sample of 29 news reports, found that 79% (95% CI 61%-90%) of reports do not extrapolate beneficial effects in humans from animal studies. ^35^ The criterion had a low risk of bias.

**Criteria related to pros and cons**

Few news reports failed to mention at least one benefit of an intervention. On the other hand, few mentioned or discussed the cost of the intervention, and most did not mention or adequately discuss or explain potential harms.

Altogether, we included 32 estimates, from 17 studies, ^12, 13, 29– 31, 35, 38– 41, 43, 47– 52^ related to IHC Key Concepts that *“Treatments can cause harms as well as benefits”* (1.1a) and *“Weigh the benefits and savings against the harms and costs of acting or not”* (3.3a). One criterion was also included with criteria related to qualitative descriptions of effects, related to the IHC Key Concept that *“Verbal descriptions of the size of effects alone can be misleading”* (2.3a). That criterion was the only one that had a high risk of bias.

We included 14 criteria, from 13 studies, ^12, 13, 30, 35, 40, 41, 43, 47– 52^ related to harms. Overall, 40% (95% CI 23%-61%) of news reports about the effects of health interventions mention or adequately discuss or explain potential harms of the intervention. The certainty of the evidence for that estimate is low, due to substantial inconsistency (I ^2^ = 99%) and important imprecision.

We included 10 criteria, from nine studies, ^13, 29, 30, 40, 41, 43, 49– 51^ related to cost. Overall, 18% (95% CI 12%-28%) mention or discuss the cost of the intervention. The certainty of the evidence for that estimate is moderate, due to substantial inconsistency (I ^2^ = 98%).

We included two criteria, from two studies, ^41, 50^ related to benefits. Overall, 97% (95% CI 56%-100%) of news reports mention at least one benefit. The certainty of the evidence for that estimate is moderate, due to imprecision.

## Discussion

### Key findings

Overall, we found that researchers have conducted many empirical studies of the quality of news reports about the effects of health interventions, assessing diverse samples of reports in terms of medium, country (although primarily English-speaking and high-income), and categories of interventions, using many different tools and criteria. Some criteria did not make sense and some relevant aspects of quality were not covered. We were able to synthesis results for some criteria groups, showing that there were several prevalent and important problems with the quality of news reports about the effects of health interventions. Many of the reports gave an unbalanced and oversimplified picture of the potential consequences of the interventions, leaving out information about conflicts of interest, alternative interventions, potential harms, and costs, and failing to quantify effects in absolute terms or at all.

Specifically, we identified 44 primary studies for inclusion in the qualitative summary (see extended data - S1 Table ^105^). Newspaper was the most common medium. Of the 39 studies that specified a medium, newspaper content was explicitly included in 35 (90%). The United States (US) was the most common country in which content was published. Of the 40 studies where at least one country was specified, 12 (30%) included content published in the US, while 25 (63%) included content published in English-speaking countries. “Modern” medicine was the most common category of health interventions. In the 32 samples where a category of intervention was specified, 24 (75%) included news reports about “modern” medicine. No study specified whether sampled reports were published by commercial or non-commercial outlets. Most studies used a tool that was original or modified.

The Index of Scientific Quality was the most common tool (set of criteria). It was used in seven studies (16%). ^31, 33, 34, 36– 39^ However, a larger number of studies used tools directly or indirectly based on the criteria developed by Moynihan *et al.*, ^40^ including the four studies that used the Media Doctor Australia tool ^12, 13, 41, 42^ and the three studies that used the Health News Review tool. ^28, 29, 43^

Of the 44 studies included in the qualitative summary, we included 18 (41%) in the meta-analyses. We mapped all 208 quality criteria used in those studies against the IHC Key Concepts. Some of the criteria were in conflict with IHC Key Concepts, such as those that asked whether research discussed in a news report was peer-reviewed. We were unable to identify or include quality criteria related to several highly relevant IHC Key Concepts, for example “ *Average differences between treatments can be misleading*” (2.3c).

In the end, we included 108 of the 208 criteria (52%) in the meta-analyses, organised in 19 groups, as well as eight subgroups. We calculated overall estimates and rated the certainty of the evidence for five of the 19 main criteria groups (26%) and 6 of the 8 subgroups (75%). Those estimates ranged from 17% of news reports satisfying criteria related to reporting absolute effects (95% CI 4%-49%) (low certainty), to 97% satisfying criteria related to reporting on benefits (95% CI 56%-100%) (moderate certainty).

### Context

**Media doctor Australia and health news review**

Some of the studies included in our review report partial results from initiatives aimed at helping journalists and the public assess health information in the news and improving health journalism. This includes studies related to the Media Doctor Australia initiative ^12, 13, 42^ and its American counterpart Health News Review (or HealthNewsReview.org). ^28, 29^ These initiatives involved an editorial team rating news reports about the effects of health interventions on a running basis and posting individual ratings with lay explanations on a dedicated website. Both have been shut down. We were unable to include the most complete published results from either initiative in our meta-analyses, but those results were consistent with the earlier results that we were able to include, as well as our overall findings.

In terms of Media Doctor Australia, we were able to include the studies by Smith *et al.* ^12^ and Bonevski *et al*. ^13^ in the meta-analyses. The former includes results from the first 104 ratings, published between Feb. 1 and Sept. 1, 2004, while the latter includes results from the first 222 ratings of news reports about “complementary” and “alternative” medicine, published between Jan. 1, 2004, and Sept. 1, 2007. Meanwhile, Wilson *et al*. include results from 1230 ratings, posted between March 2004 and June 2008, in a paper titled “Media reporting of Health Interventions: Signs of Improvement, but Major Problems Persist”. ^42^ We included the study by Wilson *et al.* in our qualitative summary, but not in the meta-analyses, since the authors only reported mean scores and not the sample sizes for each quality criterion. We knew from the two other Media Doctor Australia studies that sample sizes varied between criteria because a criterion was not always considered relevant to a given news report and, in those cases, not applied. In other words, we could not extract the exact proportions of news reports satisfying each criterion from the study by Wilson *et al*. That said, as the title of paper suggests, there were no notable differences between the new Media Doctor Australia results and the previous results reported by Smith *et al*. (see Table 2 in the study by Wilson and colleagues ^42^).

In terms of Health News Review, we were able to include the studies by Schwitzer ^28^ and Walsh-Childers *et al.* ^29^ in the qualitative summary. The former included results from 500 Health News Review ratings, published between April 2006 and February 2008, while the latter included results from 1889 ratings published from July 2005 through March 2013. Hence, we only included the study by Walsh-Childers *et al.* in the meta-analyses. The Health News Review team continued to publish ratings until the end of 2019, finishing at 2610. ^53^ In a blog post published Dec. 20, 2018, the founder and publisher, Schwitzer, shared a final “report card” with some of the results from all 2610 ratings. ^53^ Like with the Media Doctor Australia studies, there were no notable differences between the most recent results, published in Schwitzer's blog post, and those previously reported and included in this review, from the study by Walsh-Childers *et al*.

**Underlying problems**

Problems with the quality of information in health news reports could be explained by underlying problems occurring at different stages of the information pipeline, from when scientists plan, conduct and report studies, to when academic institutions and journals promote studies, to when news outlets cover studies. Not to mention deliberate misinformation by commercial industries and organisations. ^54^

Ioannidis has estimated that most published research findings are false due to small studies and small effect sizes, testing many relationships without preselecting those tests, flexibility in study designs, definitions, outcomes and analyses, financial and other conflicts of interest, and competition. ^55^ Similarly, Chalmers and Glasziou have estimated that 85% of research is wasted due to wrong research questions, unnecessary or poorly designed studies, failure to publish relevant research promptly or at all, and biased or unusable reports of research. ^56^^,^^57^ Meanwhile, Chi and colleagues have found problems with the quality of information in biomedical literature that correspond with problems found in health news reporting, for example inappropriate use of causal language. ^58^ A vast amount of poorly planned, conducted and reported research provides a terrible starting point for health and science journalism. Parenthetically, these studies provide additional evidence that peer review is a poor indicator of quality.

Several studies have identified problems with press releases about health research. Wang *et al*. found randomised trials were less likely to be the subject of a medical journal press release compared to observational studies. ^59^ Woloshin *et al.* assessed press releases from academic medical centres and found many were directly misleading or misleading by omission. ^60^ Yavchitz *et al.* found press releases for two-arm, parallel-group randomised trials often included some form of “spin”, for example conflating lack of evidence of a difference with no difference. ^47^ Sumner *et al.* have found exaggerations in press releases from both academic institutions ^61^ and scientific journals, ^62^ such as causal claims based on observational studies, were associated with exaggerations in related news reports. This finding was supported by a replication study conducted by Bratton *et al*. ^63^

Amend and Secko conducted a qualitative synthesis of studies exploring journalists’ experience of covering health and science. ^64^ They included 21 studies involving 788 journalists in total. Many of the themes cutting across studies were barriers to high-quality health and science journalism, including deadline pressure and limited time for researching and preparing stories; limited time or space for telling stories; limited budgets and staff, including a lack of specialised health and science journalists; competition and commercialization; pressure from advertisers and interest groups; and lack of education or training. More recently, O’Keeffe *et al*. found some of the same barriers to reliable reporting when they interviewed 22 Australian journalists, most of whom were specialised in health. ^65^

**Other mass media sources of health information**

Other studies have focused on other mass media sources of lay information about the effects of health interventions. This includes both other forms of journalism, besides news reports, and other mass media, besides news media. Some of those studies were included in this review. Overall, it appears quality varies depending on the source, but that there are problems across the board, granted we have not systematically reviewed or critically appraised studies focusing on other sources.

*Newspaper features and advice columns –* Heaner assessed news features and news reports about nutrition and physical activity. ^32^ She found features were generally of higher quality. Molnar *et al.* assessed medical advice columns for elderly readers and found that recommendations were often inappropriate or potentially dangerous. ^66^

*Entertainment –* Wilson *et al*. assessed the quality of advice popular magazine articles and found especially those with “health” in the title of the publication presented poor-quality, unreliable advice. ^67^ Korownyk *et al*. and Mishori *et al.* assessed recommendations made on medical talk shows. ^68, 69^ Both studies found the recommendations were often unreliable or misleading.

*Social media –* Moorhead *et al.* conducted a systematic review of the uses, benefits and limitations of social media for health communication and found that concern about the quality of information was common across studies. Li *et al.* found that over a quarter (19) of the 69 most-viewed YouTube videos about Covid-19 contained misleading information, including “inappropriate recommendations”, consistent with previous studies of YouTube videos about other pandemic diseases. ^70^ Haber *et al.* found many of the health science articles most often shared on Twitter and Facebook in 2015, as well as media coverage of those articles, often used causal language that was too strong for the strength of the evidence. ^27^

*Advertising –* There is a long tradition of misleading advertisements for health interventions. Dushman has provided 10 examples from the early 20 ^th^ century, including an ad for Dr. William O. Coffee's “world-famous”, “marvelous” and “remarkable” treatment for deafness. ^71^ Frosch *et al*. and Faerber and Kreling have found problems with modern advertisements for both prescription and over-the-counter medicines. ^72, 73^ In sales materials for herbal dietary supplements, the United States Government Accountability Office has found “improper” and illegal claims that the supplements can cure cancer, among other conditions. ^74^

*Other consumer and patient sources* – In a systematic review of studies assessing the quality of online health information for consumers, Eysenbach *et al.* found 55 of 79 studies (70%) had concluded that quality was a problem. ^75^ In more recently published studies, others have found problems with the information on anti-vaccination websites, ^76^ cancer and oncological industry websites, ^77^ websites for clinics offering weight loss surgery, ^78^ and websites for fertility centres. ^79^ In their study of media coverage of practice-changing clinical trials in oncology, Andrew *et al*. found the cancer and industry websites provided higher-quality information than newspaper and cable news reports. ^77^

Studies focusing on patient sources of information have found problems with patient health portals, ^80^ patient materials, ^81^ and brochures from a health fair. ^34^ Glenton and Oxman (AO) surveyed and interviewed leaders of organisations representing health care users. ^82^ They concluded that the organisations did not appear to promote evidence-based health care and that when they did promote scientific information, they appeared to do so uncritically, relying on limited sources and traditional authorities.

Last but not least, in a review published in 2019, Oxman and Paulsen assessed free, online sources of “trustworthy” information about the effects of health interventions. ^83^ They found that patients and the public could access trustworthy information using two websites: Cochrane Evidence ( www.cochrane.org/evidence) ^84^ and Informed Health ( www.informedhealth.org). ^85^ However, they concluded that both websites could be improved by consistently reporting information about the size of both benefits and harms, and the certainty of the evidence. They noted that many websites excluded from their review claim to provide evidence-based or reliable information about the effects of health interventions, but that it was difficult to assess the reliability of the information on those websites since the information was not explicitly based on systematic reviews.The Informed Health website is a separate initiative from the Informed Health Choices project.

**Other problems with health news**

Other studies have found other problems with health news, besides the quality of information. Wang *et al*. found that observational studies and small randomised trials reporting surrogate outcomes generated as much news coverage as large, randomised trials reporting important outcomes. ^59^ Dumas-Mallet *et al.* found that newspaper reports about biomedical research were typically about findings from initial studies. ^86^ Such findings were often contradicted by a meta-analysis, but this was rarely reported by news outlets.

**Strengths**

As far as we are aware, this is the first systematic review of the quality of any type of health news. It provides a larger, more detailed picture of the quality of health news than any primary study that we identified. It also provides a starting point for similar reviews and alternative analyses, and it highlights important issues for interpreting and planning new studies. We conducted an expansive search and identified several studies outside the peer-reviewed literature (see extended data - S1 Table ^105^). We assessed records and full texts in any language for inclusion in the review, and included studies in five different languages in the qualitative summary (English, German, Norwegian, Slovenian and Spanish) (see extended data - S1 Table ^105^). We synthesised results from up to 13 different studies, using different tools and criteria, and pooled results for up to 4116 unique news reports (see extended data - S8 and S10 Files ^105^). The review provides an accessible summary of what is known about the quality of news reports about the effects of health interventions, in print, broadcast and online news media, for those aspects of quality that have been measured and reported. It also identifies gaps in what is known and directions for future research.

**Limitations**

The findings of this review are limited to information about the effects of health interventions and “traditional” news media. In the section “Other mass media source of health information”, we reference and discuss findings from research focusing on other news media and other non-news media, but we have not systematically reviewed or critically appraised such studies. Furthermore, we did not explore applicability of the synthesised results to different types of traditional news media due to limited sample sizes, as well as inadequate reporting in the included studies.

Examples of other types of health information include the accuracy of diagnostic tests and information about the health effects of exposures. News reports about the health effects of exposures (e.g. fatty foods) can be difficult to distinguish from news reports about the effects of health interventions (e.g. a low-fat diet). Some primary studies included in this review conflated the two, such as the study by Hackman and Moe, which assessed news about nutrition-related research. ^87^ We included this study in the qualitative summary, but not the meta-analyses. We did this because key concepts for assessing or communicating information about the effects of interventions may not apply to the same degree, or at all, to information about associations between exposures and outcomes. For example, Woodruff and Sutton point out that randomised trials are “virtually precluded” from evidence about hazardous environmental exposures because of ethical considerations”. ^88^ It is possible, if not likely, that there are important differences in quality between news reports about the effects of exposures versus interventions.

We excluded studies or criteria that were only descriptive, even though criteria that were descriptive sometimes provided the same data as criteria explicitly intended to assess quality. For example, Mercurio and Eliott describe Australian news about “alternative” medicine for cancer, but do not explicitly assess the quality of those news reports, ^89^ so we excluded the study. However, one of their coding variables was “Mention of risks, benefits and costs”. The data for this variable would have fit with data in our meta-analyses, in the group Criteria related to pros and cons. We consider it unlikely that inclusion of studies or criteria that were only descriptive would have substantially altered the findings of this review. Similarly, we only included studies in the meta-analyses if they were in English or Norwegian (one of the languages spoken fluently by the primary investigator, MO), but consider it unlikely that the studies reported in other languages would have substantially altered the findings of this review either, given that the underlying problems discussed earlier on are unrelated to language.

We did not code raw data ourselves or contact authors for help where data were inadequately reported for our purposes. Finally, we did not consider which criteria were more informative, only whether they were eligible and sensible, nor did we consider statistical measurement properties. ^90^

### Implications

Implications for different stakeholders are summarised in Table 5 and explained in more detail in the subsequent sections.

**Table 5.** Summary of implications.

Stakeholder		Implications
*All*		• Pay attention to relevant IHC Key Concepts
*Journalism*		
• Journalists		• Approach research and press releases critically • Search Cochrane Evidence and Informed Evidence for relevant summaries
• Editors • News outlets		• Train health and science journalists • Provide adequate conditions for researching, preparing and telling stories
*Research*		
• Researchers • Journals • Academic institutions • Funders	General	• Reduce false research findings and research waste
• Investigators • Peer reviewers	Quality of health news	• Think carefully about the goal, tools and quality criteria • Consider applicability, explanatory factors and risk of bias • Be explicit, clear and comprehensive in reporting
*Consumers and patients*		• Approach health news critically • Search Cochrane Evidence and Informed Evidence for relevant summaries
*Citizens and policymakers*		• Advocate for teaching IHC Key Concepts in school

**Implications for journalists, editors, and news outlets**

There is clearly room for improving the quality of health and science journalism. Based on our meta-analyses, journalists and editors should pay special attention to the following IHC Key Concepts (see extended data - S6 File ^105^) when communicating information about the effects of health interventions:

• “Treatments can cause harms as well as benefits” (1.1a),
• “Competing interests may result in misleading claims” (1.3b),
• “Verbal descriptions of the size of effects alone can be misleading” (2.3a),
• “Relative effects of treatments alone can be misleading” (2.3b),
• “Be clear about what the problem or goal is and what the options are” (3.1a), and
• “Weigh the benefits and savings against the harms and costs of acting or not” (3.3a).

Based on individual estimates from primary studies included in this review (see extended data - S8 and S10 Files ^105^), there is likely also room for improvement related to other highly relevant IHC Key Concepts, such as “ *The treatments compared should be similar to those of interest*” (3.2c). Moreover, there is likely room for improvement related to highly relevant IHC Key Concepts that were not captured by any quality criteria, such as “ *Average differences between treatments can be misleading*” (2.3c) (see extended data - S10 File ^105^).

Journalists and editors should take a critical approach to reports of research, especially single studies and observational studies, and to press releases (see “Underlying problems”). When reporting on the effects of interventions, they can search the Cochrane Evidence ( www.cochrane.org/evidence) ^84^ and Informed Health ( www.informedhealth.org) ^85^ websites for summaries of relevant systematic reviews. If possible, news outlets should provide or support special training for health and science reporters, as well as provide adequate time for researching and preparing stories, and adequate time or space to tell stories.

Journalists and editors who are interested in learning more about IHC Key Concepts can do so for free on the “That's a claim!” website ( www.thatsaclaim.org), ^91^ where all of the concepts are explained in plain language, and there are references to relevant learning resources for each individual concept. They can also find free learning resources for respective concepts via the Teachers of Evidence-Based Health Care portal ( www.teachingebhc.org). ^92^

**Implications for researchers, scientific journals, academic institutions, and research funders**

Researchers, journals, and academic institutions should pay special attention to the same IHC Key Concepts as journalists and editors, as well as other highly relevant concepts (see previous section), when reporting the results of studies assessing the effects of interventions in research papers and press releases, as well other contexts, such as conferences. More fundamentally, they should work to reduce false findings and research waste. This also goes for organisations that fund research.

The results of this review have a series of implications for investigators planning new studies of the quality of information about the effects of health interventions in mass media (as well as peer reviewers assessing new studies), from objectives, to methods, to reporting. First, they should think carefully about their goal. New studies can be designed to have practical value, for example informing interventions to improve a particular population's ability to think critically about health information by identifying the IHC Key Concepts that are most relevant for that population—e.g. a study of health claims in advertisements targeted at young people. In addition, they should consider research gaps that we have revealed. Again, there does not appear to be studies assessing the quality of health news reports in terms of several highly relevant IHC Key Concepts (see extended data - S6 Table ^105^). Neither does there appear to be studies that compare the quality of reports published by commercial outlets versus public or independent outlets. Others have pointed out that research is needed about how best to communicate information about the effects of health interventions, ^93^ including how to communicate uncertainty. ^94^

Second, they should think carefully about what tools and quality criteria to use, whether using a previously developed tool, adapting one, developing a new tool, or some combination. They should avoid criteria that are overly general (e.g. global scores and composites) or unclear (e.g. criteria assessing whether “double blinding” is mentioned), and criteria that do not make sense (e.g. criteria assessing whether research was peer-reviewed). They should be deliberate about the trade-offs between how straightforward it is to apply a criterion and report the results versus how informative it is. For example, it is relatively easy to see whether a news report mentions the design of a study. It is more difficult, but also more informative, to assess whether the report adequately discusses strengths and limitations of said design.

Third, regarding samples of news reports, they should consider applicability and potential explanatory factors, for example those we highlighted in the protocol for this review (see extended data - S1 File ^105^) as possible variables for subgroup analyses: medium; time period; financial model (commercial vs. non-commercial); specialisation (specialised health or science journalist vs. other); country income level; and newspaper type (broadsheet vs. tabloid). Fourth, they should consider risk of bias. If possible, two researchers should assess each news report or other unit of mass media content, and the researchers should be blinded to factors that could introduce bias, such as the name of the journalist or publication. This is especially important for criteria that require substantial judgement.

Fifth, when reporting new studies, researchers should be explicit, clear and comprehensive. They should include the study objective, the sampling frame, and the selection criteria and technique, with concern for applicability and comparing results across studies, including results for subgroups. They should include the time period in which the content was published, as well as when it was assessed; the country in which it was published or, if online content, the country that the outlet primarily targets or where it is based; the categories of interventions that the content focuses on; and the financial models of the outlets. If using a previously developed tool, they should provide reference to where the development is described, note whether they made any adjustments and, if so, explain them. If using a new tool, they should clearly describe how it was developed and what informed it, possibly in a separate paper. If the researchers received any training in applying the criteria, they should describe what this entailed. Finally, they should report results for each quality criterion separately, not just mean scores, and report absolute numbers (the total number of news reports or other units to which the criterion was applied and the number of units that satisfied the criterion), including for subgroups.

**Implications for consumers and patients**

The implications for consumers and patients are similar to those for journalists and editors. They should pay special attention to the same IHC Key Concepts, as well as other highly relevant concepts (see “Implications for journalists”); they should approach health information in the mass media critically; they can refer to the Informed Health and Cochrane Evidence websites for summaries of relevant systematic reviews; and they can take advantage of the “That's a claim!” website and Teachers of Evidence-Based Health Care portal to learn more about IHC Key Concepts.

**Implications for citizens and policymakers**

The results of this review, as well as the results of related studies referenced in the section “Other mass media sources of health information”, show that the global “infodemic” was compounded by Covid-19, but started long before the World Health Organization coined the term in their situation report on the virus published Feb. 13, 2020. ^3^ The infodemic is so dangerous because we know so many people are unable to critically assess claims or evidence about the effects of health interventions. ^95, 96^ When people act on those claims or beliefs that are unreliable or fail to act on reliable advice, they may suffer unnecessarily or waste resources. It follows that infodemic is probably an important factor in the enormous, worldwide overuse of ineffective or harmful interventions ^97^ and underuse of effective interventions. ^98^ Even one misleading news report might contribute to wasteful, harmful, and even deadly choices. A dramatic example of misleading health news is coverage of the baseless suggestion that the measles, mumps and rubella vaccine causes autism. ^99^ Incidentally, news reports often focus on the benefits of interventions while ignoring or downplaying the harms, but this example illustrates that unbalanced reporting can also go in the opposite direction.

If the only goal of news about the effects of health interventions was to facilitate well-informed health choices, the content and format might look more like Cochrane plain language summaries, which have been systematically developed towards exactly said end. ^100, 101^ The reality is that news outlets existentially depend on getting people's attention, not informing them. Commercial media rely on advertising, not least advertisements for health interventions, which provides incentive to sensationalise. In turn, this can create pressure on competing public and independent outlets to do the same. Along the same lines, the raison d'être of press releases is inciting media coverage, while scientists are forced to “publish or perish”. Setting aside systemic barriers to reliable health news (see the section “Underlying problems”), it may be difficult if not impossible for journalists to produce news reports that satisfy all sensible, applicable criteria identified in this review in a report that people would understand and want to read, not to mention other potential criteria related to other highly relevant IHC Key Concepts. Even if a report includes and appropriately presents all relevant information about an intervention, it is possible that many people will not read more than the headline, missing important details, such as potential side effects.

With enough resources, it is possible for news outlets, including commercial ones, to improve their health and science coverage without shrinking their audiences and perhaps even increasing their numbers of readers, listeners, or viewers in some cases. But putting an end to unreliable health news is a Sisyphean task, not to mention health misinformation from other sources. In 2009, during a debate titled “Does the media support or sabotage health?”, Goldacre suggested: “The future of people getting information about health does not lie in journalism but people having direct access to information or the world of blogs where people can link direct to primary sources. There is no role for the health journalists of the future other than as entertainers". ^102^ However, it seems unlikely that giving people more health information—even if it is reliable—will solve the problem, when they are already overwhelmed and lack the skills needed to “separate the wheat from the chaff”.

As the saying goes, give someone a fish and you will feed them for a day; teach them to fish and you feed them for a lifetime. In other words, you can provide people with reliable health information or advice, and maybe they will believe you. If you help them understand why it is reliable, or why a claim is unreliable, you are preparing them to assess information and make informed choices for themselves in the future, inoculating them against unreliable claims and uninformed choices. Hence, we should be helping people gain basic critical appraisal and decision-making skills, in addition to providing access to reliable information. Moreover, by improving people's ability to assess evidence or recognise a lack of reliable evidence, the public may start to demand reliable studies, reducing false findings and research waste.

Focusing on the education system allows for reaching large groups of people, gathered specifically to learn. It is important to start young, when people have time to reinforce and build upon what they have learned, and they have less to unlearn. The IHC network, which includes authors of this review, has developed respective interventions to help primary school children ^103^ and university students ^104^ master select IHC Key Concepts.

## Data availability

### Underlying data

All data underlying the results are available as part of the article and its supporting information files and tables.

### Extended data

Zenodo: Quality of news reports about the effects of health interventions - Supporting Information. http://doi.org/10.5281/zenodo.4781182. ^105^

This project contains the following extended data:

- **S1 File. Protocol.** “Quality of news media reports about the effects and costs of health interventions: Systematic review protocol”.
- **S2 File. Protocol Appendix 1.** “Appendix 1: Reference list of potentially eligible studies”.
- **S3 File. Protocol Appendix 2.** “Appendix 2: Description of search for an existing review”.
- **S4 File. Dissertation.** “Criteria used to measure the quality of news media reports about the effects of health interventions: Systematic review”.
- **S5 File. Detailed search strategy.**
- **S6 File. Informed Health Choices Key Concepts.** 2019 version.
- **S7 File. Detailed information about deviations from the protocol.**
- **S8 File. Forest plots for individual criteria groups.**
- **S9 File. Individual risk of bias assessments.**
- **S10 File. Individual criteria included in meta-analyses.** Sample characteristics, verbatim and reworded criteria, related IHC Key Concepts, overall risk of bias, estimates and confidence intervals, and sample sizes.
- **S1 Table. Included studies**. References for all studies included in qualitative summary and reason for exclusion from meta-analyses or number of criteria included in meta-analyses.
- **S2 Table. Sample characteristics and tools by study.** Sample size, medium/media, country/countries, time period(s), intervention category/categories, and tool(s).
- **S3 Table. Objectives of included studies.**
- **S4 Table. Sampling frames and methods.**
- **S5 Table. Reported subgroup analyses.**
- **S6 Table. Number of quality criteria included in the meta-analysis per relevant IHC Key Concept.**

### Reporting guidelines

Zenodo: PRISMA checklist for ‘Quality of information in news media reports about the effects of health interventions: systematic review and meta-analyses’. http://doi.org/10.5281/zenodo.4781182. ^105^

Data are available under the terms of the Creative Commons Attribution 4.0 International license (CC-BY 4.0).

## Author contributions

MO drafted the protocol and this paper. AO provided feedback on the draft protocol. All co-authors provided feedback on drafts of this paper. MO, LL and AQ piloted the eligibility criteria for primary studies. MO conducted all database searches, including citation searches. MO, LL, GG, DA, AQ and KB checked reference lists. All authors apart from CR contributed to study selection. All authors apart from AO and CR contributed to data extraction. MO and AO assessed, mapped and grouped the quality criteria. CR conducted the meta-analyses. MO and LL assessed risk of bias. MO and AO rated the certainty of the evidence.
