# Supplementary material for: Quality of information in news media reports about the effects of health interventions: Systematic review and meta-analyses
Source: F1000Res. 2022 Jan 31;10:433. Originally published 2021 Jun 1. [Version 2] doi: 10.12688/f1000research.52894.2 (PMC8756300; doi:10.12688/f1000research.52894.2)
Supplement: Peer review history

# Peer review: Reviewer response for version 2

**DOI:** 10.5256/f1000research.120649.r121671

**Published:** 2022-02-23

**Authors:** David Henry

**Affiliations:** 1 Institute for Evidence-Based Healthcare, Bond University, Gold Coast, Australia

My only remaining concern is the way the conclusion is framed: "it is logically more important to improve the public’s ability to critically appraise health information and make judgements for themselves". While education is preferable to regulation I don't think that improving media coverage of health is an alternative to educating the public to be more critical of health claims. We should be trying to encourage both.

Are the rationale for, and objectives of, the Systematic Review clearly stated?

Yes

Is the statistical analysis and its interpretation appropriate?

Partly

Are sufficient details of the methods and analysis provided to allow replication by others?

Yes

Are the conclusions drawn adequately supported by the results presented in the review?

Partly

Reviewer Expertise:

clinical epidemiology, pharmacoepidemiology

I confirm that I have read this submission and believe that I have an appropriate level of expertise to confirm that it is of an acceptable scientific standard.

# Peer review: Reviewer response for version 1

**DOI:** 10.5256/f1000research.56222.r90284

**Published:** 2021-08-23

**Authors:** David Henry

**Affiliations:** 1 Institute for Evidence-Based Healthcare, Bond University, Gold Coast, Australia

The other reviewers of this article have highlighted its considerable strengths and I won’t repeat their comments. This is a solid attempt to summarise studies that have made structured attempts to quantify and report key characteristics of medical news stories that report therapeutic claims. It is very useful to have this summary.

 Many of these studies were carried out to highlight the weaknesses in the reporting of medical advances in the mainstream media in the hope that this would raise standards. But there is no strong evidence that this has resulted from these efforts.

 The studies included in this review preceded the news coverage of COVID-19, its preventive and therapeutic interventions. It would be very instructive to measure how the media performed during the epidemic and whether reporting evolved. My informal impression is that respect for science, and possibly the reports themselves, have improved. However, this has been countered by ignorant and some willfully and politically motivated inaccurate reporting in a few mainstream outlets and social media. My point is that the media landscape may have changed because of Covid-19.

 I only have a few specific comments:

1. I don’t think that conventional meta-analysis produces meaningful data in this situation. Meta-analysis is at its best when we can assume that there is an underlying single effect of interest that is measured imprecisely by individual studies. For instance, the relative effects of many important medications (e.g, statins, anticoagulants), which seem constant across different settings and background risks. Meta-estimates of prevalence are less helpful as there is usually an assumed variation. That is the case here where the study methods, story topics, settings, and reporters vary. There is no reason to assume a common effect to be meta-analysed. Some of the I ^2^ values are huge. In my view it would be preferable to present more as a scoping review, displaying and discussing the range of values reported by the studies.
2. The investigators, appropriately, have mapped the varying scales and items to a common framework. They have used their own Informed Health Choices (IHC) Key Concepts framework. That’s fine, but the paper seems to assume a familiarity with the framework that many readers will not possess. I think a simplified version of the framework needs to be presented in the main text. At present, it seems to be buried in Supplementary data.
3. In the Abstract, the authors conclude: ‘There is room for improving health news, but it is logically more important to improve the public’s ability to critically appraise health information and make judgements for themselves.’ The paper does not provide data to support this conclusion. The authors have done important ground-breaking work with IHC but the extensive coverage they provide themselves in the Discussion of an already long paper seems excessive. A separate editorial/opinion piece making these points would be welcome.
4. I think there may be a mistake in the dot point summary in the Discussion which states ‘• 86% of news reports commit “disease mongering” (95% CI 78%-91%) (low certainty).’ In the paper they state ‘Overall, 86% of news reports do not commit disease mongering (95% CI 78%-91%)’. I think the latter is correct.

Are the rationale for, and objectives of, the Systematic Review clearly stated?

Yes

Is the statistical analysis and its interpretation appropriate?

Partly

Are sufficient details of the methods and analysis provided to allow replication by others?

Yes

Are the conclusions drawn adequately supported by the results presented in the review?

Partly

Reviewer Expertise:

clinical epidemiology, pharmacoepidemiology

I confirm that I have read this submission and believe that I have an appropriate level of expertise to confirm that it is of an acceptable scientific standard, however I have significant reservations, as outlined above.

# Response

**Published:** 2022-02-01

**Authors:** Matt Oxman

**Affiliations:** Norwegian Institute of Public Health, Norway

We are grateful to you and the other reviewers for your time, effort, and constructive criticism. Thank you! We have pasted your comments below, in italics, with responses in turn.

*1. I don’t think that conventional meta-analysis produces meaningful data in this situation. Meta-analysis is at its best when we can assume that there is an underlying single effect of interest that is measured imprecisely by individual studies. For instance, the relative effects of many important medications (e.g, statins, anticoagulants), which seem constant across different settings and background risks. Meta-estimates of prevalence are less helpful as there is usually an assumed variation. That is the case here where the study methods, story topics, settings, and reporters vary. There is no reason to assume a common effect to be meta-analysed. Some of the I2 values are huge. In my view it would be preferable to present more as a scoping review, displaying and discussing the range of values reported by the studies.*

 We assume that this comment is suggesting the studies were too heterogeneous for estimating average proportions across studies to be meaningful. We were conservative about synthesising results this way, and cautious in our interpretation. We used a random effects analysis, which explicitly assumes there is not a common effect, but rather that the studies estimate distinct effect sizes. The large I2 values are a property of the literature included. The range and results for individual studies are available in the supplementary material, with the forest plots. We elected to exclude this information from the text because it would make the text harder to read, and it would not substantially alter the results—i.e., despite substantial heterogeneity, the overall estimates and confidence intervals provide an appropriate indication of what was found across the studies in each meta-analysis, and a better indication than the median and range, or range alone.

*2. The investigators, appropriately, have mapped the varying scales and items to a common framework. They have used their own Informed Health Choices (IHC) Key Concepts framework. That’s fine, but the paper seems to assume a familiarity with the framework that many readers will not possess. I think a simplified version of the framework needs to be presented in the main text. At present, it seems to be buried in Supplementary data.*

 The article is already long, as pointed out in Reviews 1 and 3. We have included the framework as S6 File and reference the file at first mention of the framework in the methods section, where we also summarise its content, and explain why and how we used it.

*3. In the Abstract, the authors conclude: ‘There is room for improving health news, but it is logically more important to improve the public’s ability to critically appraise health information and make judgements for themselves.’ The paper does not provide data to support this conclusion. The authors have done important ground-breaking work with IHC but the extensive coverage they provide themselves in the Discussion of an already long paper seems excessive. A separate editorial/opinion piece making these points would be welcome.*

 We have removed the last three paragraphs of the discussion, which were about IHC.

*4. I think there may be a mistake in the dot point summary in the Discussion which states ‘• 86% of news reports commit “disease mongering” (95% CI 78%-91%) (low certainty).’ In the paper they state ‘Overall, 86% of news reports do not commit disease mongering (95% CI 78%-91%)’. I think the latter is correct.*

 Correct. We have fixed this typo.

 Thank you again for the valuable feedback!

# Peer review: Reviewer response for version 1

**DOI:** 10.5256/f1000research.56222.r90288

**Published:** 2021-08-09

**Authors:** Amanda Wilson

**Affiliations:** 1 School of Nursing and Midwifery, University of Technology Sydney, Sydney, NSW, Australia

This is a systematic review of studies assessing the quality of health news articles which provides a meta-analysis of the results. This is an interesting paper which provides a quantitative approach to what is likely to be considered a difficult area to quantify – that is quality of reporting. The influence of news reporting on health behaviours is well known and the current climate of pandemic and vaccination information makes this a highly pertinent area to explore.

 The methodology is sound and rigorous with the authors following the gold standard approach of registering the review and publishing the protocol. All steps of the literature review are described in detail and the analyses and interpretation are sound.

 The paper is extremely well written and I have little critical feedback on the structure of the research or presentation of the paper. My only criticism is that it is extremely long and possibly overly detailed. The length and detail are worthy of a Cochrane review but without the saving grace of a plain language summary. I wonder if some ruthless editing might enhance the number of readers who make it the whole way through. It feels like a PhD chapter rather than a journal article. Having said that, in the Discussion on page 18, I was interested to know which criteria did not make sense and which aspects of quality were not covered.

Are the rationale for, and objectives of, the Systematic Review clearly stated?

Yes

Is the statistical analysis and its interpretation appropriate?

Yes

Are sufficient details of the methods and analysis provided to allow replication by others?

Yes

Are the conclusions drawn adequately supported by the results presented in the review?

Yes

Reviewer Expertise:

Public health, health literacy, health reporting in the media, health education

I confirm that I have read this submission and believe that I have an appropriate level of expertise to confirm that it is of an acceptable scientific standard.

# Response

**Published:** 2022-02-01

**Authors:** Matt Oxman

**Affiliations:** Norwegian Institute of Public Health, Norway

We are grateful to you and the other reviewers for your time, effort, and constructive criticism. Thank you! We have pasted your comments below, in italics, with responses in turn.

*My only criticism is that it is extremely long and possibly overly detailed. The length and detail are worthy of a Cochrane review but without the saving grace of a plain language summary. I wonder if some ruthless editing might enhance the number of readers who make it the whole way through. It feels like a PhD chapter rather than a journal article.*

 Please see our response to Review 1. 

*Having said that, in the Discussion on page 18, I was interested to know which criteria did not make sense and which aspects of quality were not covered.*

 Regarding criteria that did not make sense, some conflict with IHC Key Concepts, such as the example in Table 3, or criteria that conflate a “statistically significant” effect with an important effect, including respective criteria used by Haneef et al. and Yavchitz et al. Others are imprecise measures of quality, for example criteria focusing on the novelty of the health intervention, or how much of a news report was based on a press release, since a report can be low or high-quality regardless of these characteristics. 

 Regarding the aspects of quality not covered, S6 Table shows which IHC Key Concepts were not related to any criteria included in the meta-analyses.

 Thank you again for the valuable feedback!

# Peer review: Reviewer response for version 1

**DOI:** 10.5256/f1000research.56222.r90763

**Published:** 2021-08-04

**Authors:** Joel R. Lexchin

**Affiliations:** 1 School of Health Policy & Management, Faculty of Health, York University, Toronto, Canada; 2 Department of Family and Community Medicine, Faculty of Medicine, University of Toronto, Toronto, Canada; 3 Department of Emergency Medicine, University Health Network, Toronto, Canada

This is an extremely comprehensive and well-done systematic review on an important topic. The authors have obviously put an enormous amount of effort into the project and the results show that. The study is well written and easy to follow. However, there are some areas where I believe that improvements can be made.

  

1. There is a potentially relevant article that the authors appear to have overlooked: Cassels *et al*. Open Medicine 2008;2(1):e20–23. Another study (Cassels *et al.,* 2003) is a longer version of one that the authors use – reference 48.
2. Page 3: The authors use a 2009 paper to claim that most people get health information either directly or indirectly from traditional news media as opposed to material that appears only on social media. However, studies that evaluate information sourced from the two different types of media may no longer be relevant to the present-day environment.
3. Page 18: The authors say that their findings come from a diverse range of countries but don’t they mean primarily western countries?
4. Page 20: The length of a newspaper story is not necessarily a measure of quality. Cassels *et al.* have shown that there are few significant differences in the overall quality between short news briefs and longer stories. (Cassels *et al*., 2003)
5. Page 22: The recommendations for improving the reporting of journalists, editors and news outlets at least partially assume the existence of resources and time to educate journalists and editors about science journalism. However, the authors do not assess whether the necessary resources are actually present and available especially in smaller media outlets.
6. Page 22: Informed Health is only available in English and German. Therefore, it is limited in terms of usefulness to those speaking other languages. Cochrane Evidence is superior in that regard.
7. Page 23 (last line): Is it Informed Choices or Informed Health? Are these the same or different?
8. Page 24: Earlier the authors refer to Informed Health and Informed Choices. Now there is a third name Informed Health Choices. Are all three the same?
9. Page 24: Have any of the educational interventions that the authors mention (references 99-104) looked at the long-term (e.g., more than 1 year) effects?

Are the rationale for, and objectives of, the Systematic Review clearly stated?

Yes

Is the statistical analysis and its interpretation appropriate?

Yes

Are sufficient details of the methods and analysis provided to allow replication by others?

Yes

Are the conclusions drawn adequately supported by the results presented in the review?

Yes

Reviewer Expertise:

Pharmaceutical policy, intellectual property rights

I confirm that I have read this submission and believe that I have an appropriate level of expertise to confirm that it is of an acceptable scientific standard.

## References

[1] : How well do Canadian media outlets convey medical treatment information?: Initial findings from a year and a half of media monitoring by Media Doctor Canada. *Open Med* .2008;2(2) :e45-8 21602940PMC3090174

[2] : Drugs in the News:How well do Canadian newspapers report the good,the bad and the ugly of new prescription drugs?. *Canadian Centre for Policy Alternatives* .2003; Reference source

# Response

**Published:** 2022-02-01

**Authors:** Matt Oxman

**Affiliations:** Norwegian Institute of Public Health, Norway

We are grateful to you and the other reviewers for your time, effort, and constructive criticism. Thank you! We have pasted your comments below, in italics, with responses in turn.

*1. There is a potentially relevant article that the authors appear to have overlooked: Cassels et al. Open Medicine 2008;2(1):e20–23.*

 We excluded Cassels et al. 2008 based on the fourth eligibility criterion, specifically for inadequate specification of the selection criteria for news reports and missing specification of the selection technique.

*Another study (Cassels et al., 2003) is a longer version of one that the authors use – reference 48.*

 We assume you are referring to the paper “Drugs in the News: How well do Canadian newspapers report the good, the bad and the ugly of new prescription drugs?” published by the Canadian Centre for Policy Alternatives. We were unaware of this version. However, it does not include data that we were missing from the shorter version. 

*2. Page 3: The authors use a 2009 paper to claim that most people get health information either directly or indirectly from traditional news media as opposed to material that appears only on social media. However, studies that evaluate information sourced from the two different types of media may no longer be relevant to the present-day environment.*

 Our claim is that “people still get health information from the [news media],” not that most people get information directly or indirectly from traditional news media. We agree that distinguishing news and social media is challenging, and not necessarily sensible, which is an important point for new primary studies of the quality of health information in mass media.

*3. Page 18: The authors say that their findings come from a diverse range of countries but don’t they mean primarily western countries?*

 Yes, except we mean English-speaking and high-income countries, not Western. We have specified this. 

*4. Page 20: The length of a newspaper story is not necessarily a measure of quality. Cassels et al. have shown that there are few significant differences in the overall quality between short news briefs and longer stories. (Cassels et al., 2003)*

 We agree and we have removed the second clause of the sentence, which was a possible explanation for the finding that features were generally of higher quality than news reports. 

*5. Page 22: The recommendations for improving the reporting of journalists, editors and news outlets at least partially assume the existence of resources and time to educate journalists and editors about science journalism. However, the authors do not assess whether the necessary resources are actually present and available especially in smaller media outlets.*

 In the section “Implications for citizens and policymakers”, we now specify that news outlets can improve their reporting “with enough resources”. In the section “Implications for journalists, editors, and news outlets”, we had already specified that news outlets should provide or support training, and provide adequate time and resources, “if possible”. And in the section “Underlying problems”, we had already pointed out that in their synthesis, Amend and Secko found limited time or space for telling stories, and limited budgets and staff were barriers to high-quality health and science journalism. 

 In fact, we assume most outlets either cannot provide training or resources to improve their health and science journalism, or will not prioritise such spending, which is part of why we argue that it is so important to help all people, not just journalists, learn how to think critically about health information for themselves. That said, we already referred to free learning resources that journalists can use to improve their skills, without necessarily spending a lot of time. 

*6. Page 22: Informed Health is only available in English and German. Therefore, it is limited in terms of usefulness to those speaking other languages. Cochrane Evidence is superior in that regard.*

 We agree, but it is still helpful to be aware of both sites.

*7. Page 23 (last line): Is it Informed Choices or Informed Health? Are these the same or different?*

 It is “Informed Health”. We have corrected the typo.

*8. Page 24: Earlier the authors refer to Informed Health and Informed Choices. Now there is a third name Informed Health Choices. Are all three the same?*

 At first mention of the Informed Health website, we now note that it is a separate initiative from the Informed Health Choices (IHC) project. Again, where we have referred to the site as “Informed Choices”, it should have said “Informed Health”, and we have fixed this. 

*9. Page 24: Have any of the educational interventions that the authors mention (references 99-104) looked at the long-term (e.g., more than 1 year) effects?*

 No, and we believe that a series of interventions is necessary, reinforcing and building on what people have learned. However, this has not been tested. Note that we have removed the last three paragraphs, in response to Review 1.

 Thank you again for the valuable feedback!

# Peer review: Reviewer response for version 1

**DOI:** 10.5256/f1000research.56222.r89219

**Published:** 2021-07-29

**Authors:** Raymond Moynihan

**Affiliations:** 1 Institute for Evidence-Based Healthcare, Faculty of Health Sciences and Medicine, Bond University, Gold Coast, Australia

**General Comments.**

 This review is extremely valuable – the first summarizing evidence about the quality of news reports about health interventions.

 I have a few short general comments and some specific comments.

 In relation to the use of the word  “qualitative” - I wonder if the authors might consider using a different word – or adding more explanation – perhaps saying “qualitative summary of results”  – as it sounds to the casual reader like when you refer to “qualitative” you may have done a SR of “qualitative” studies of news coverage (i.e. focus groups with journalists etc.) – whereas I think you have a different meaning in mind. (e.g.  P8 “qualitative summary”, which is also mentioned in Abstract).

 Re: IHC. Given the importance of IHC concepts for this SR, and the role of the authors in IHC world, and the advocacy of IHC in the Discussion, I think the IHC concepts etc. needs to be mentioned in the Introduction and expanded on in the Methods (as to how and why exactly the IHC concepts interacted with your analysis in the SR) - and the authors link to IHC can be mentioned in Methods somewhere. This is important and will help explain how the Discussion section becomes a little like advocacy for IHC materials.

 The paper is long and complex – but with much valuable information. If any more material can be moved into Supp files, that will be helpful.

 I have no biostats training so I have not assessed the stats.

**Specific comments.**

- Search strategy: It seems almost 2 years since it was updated. Authors might consider updating before indexing.
- P5:  “We excluded inappropriate criteria…’   Perhaps “ineligible” might be better?
- P7: Re: your sentences “Furthermore, we reframed negative quality criteria as positive, and paraphrased all quality criteria, for the data to be consistently framed and worded, and for the criteria to be succinctly labelled. For example, Bubela et al. report the number of news reports that “did not” specify whether a study was randomized, which was reframed as the number of news reports that did specify whether a study was randomized.”  
  While this makes sense, I presume that sometimes that re-framing was not straight forward – and I wonder if there is anything more to say on any complexities associated with this approach. Also, if you can give a little more detail in the Bubela example, with numbers, that could help the reader.
- P7: “Risk of Bias”. Can the authors add a few words in text explaining why the "risk of bias" was solely about the risk of detection bias? A reader of an SR might normally expect a few different domains, so it would be good to just explain why this single domain was most relevant.
- P10: Table 2. I think Table 2 needs re-thinking – as it took me some time to work out what was going on. I was treating each row as a discreet coherent entity, but in fact you have multiple parts to each row. I am sure you can make it clearer somehow.
- P11: “In contrast, a checklist for people communicating evidence-based information about the effects of healthcare interventions includes the item “Help your audience to avoid misinterpreting continuous outcome measures,” granted the checklist is not for journalists.” I don’t fully understand meaning of this sentence - perhaps be a little clearer?
- P12: This is a structural suggestion. By the sub-heading “Synthesis of Results” I am really wanting to hear some of the key findings – in terms of content (i.e. a strong compelling prose version of the data in Figure 3)  – but I feel I am still getting a lot of complex fine detail about the characteristics of studies and elements of them. I understand there is a lot to report – but some clear statements of the key findings – even summarised and abstracted a little – would be helpful around here to orient the reader.
  Also – a related point - I find the three paragraphs on P12 (and into P13) complex and hard to follow. I wonder whether this level of detail is necessary in the main text – and whether it might be moved to a supplementary – but that is for authors to decide. At the very least – if there is any way to make it all a little clearer around this point – that would be great for the reader. An example of the mention of the “ninth criteria” from Bubela and 12 ^th^ from Haneef.  I think this is way too much detail for main text and authors run the risk of losing readers, for what is a very valuable SR.  
  Now I have read further I realise you have a long section  - starting on P14 – on the results – as they relate to IHC concepts. After having read that very long section, I still feel the need for some kind of punchy summary of the key findings (e.g. harms, COIs, costs, other options not often mentioned…) somewhere in the results section.  This kind of summary doesn’t appear until start of Discussion – but I think could be included earlier.   
- P18: I wonder if the dot points at bottom of P18 might be better in the Results – and help to drive the kind of summary I am suggesting above.
- Discussion: the paper feels like it morphs in the Discussion into a passionate advocacy for IHC materials – with a sometimes moralistic tone. I think the tone could be addressed. And I also think that in order to justify the IHC material in the Discussion, more needs to be said about IHC in the Introduction – and the authors relations with IHC need to be made more explicit in the Methods – as noted above.  

Are the rationale for, and objectives of, the Systematic Review clearly stated?

Yes

Is the statistical analysis and its interpretation appropriate?

I cannot comment. A qualified statistician is required.

Are sufficient details of the methods and analysis provided to allow replication by others?

Yes

Are the conclusions drawn adequately supported by the results presented in the review?

Partly

Reviewer Expertise:

Medicine and Media; Conflicts of Interest; Overdiagnosis

I confirm that I have read this submission and believe that I have an appropriate level of expertise to confirm that it is of an acceptable scientific standard.

# Response

**Published:** 2022-02-01

**Authors:** Matt Oxman

**Affiliations:** Norwegian Institute of Public Health, Norway

We are grateful to you and the other reviewers for your time, effort, and constructive criticism. Thank you! We have pasted your comments below, in italics, with responses in turn.

*In relation to the use of the word  “qualitative” - I wonder if the authors might consider using a different word – or adding more explanation – perhaps saying “qualitative summary of results”  – as it sounds to the casual reader like when you refer to “qualitative” you may have done a SR of “qualitative” studies of news coverage (i.e. focus groups with journalists etc.) – whereas I think you have a different meaning in mind. (e.g.  P8 “qualitative summary”, which is also mentioned in Abstract).*

 We have replaced “qualitative summary” with “review” in the abstract. And at first mention of “qualitative summary” in the main text, we explain that it is, not a synthesis of qualitative studies, but a structured synthesis of results excluded from the meta-analyses.

*Re: IHC. Given the importance of IHC concepts for this SR, and the role of the authors in IHC world, and the advocacy of IHC in the Discussion, I think the IHC concepts etc. needs to be mentioned in the Introduction and expanded on in the Methods (as to how and why exactly the IHC concepts interacted with your analysis in the SR) - and the authors link to IHC can be mentioned in Methods somewhere. This is important and will help explain how the Discussion section becomes a little like advocacy for IHC materials.*

 We have added a paragraph about IHC to the end of the introduction, where we mention the IHC Key Concepts and the relevant authors’ involvement in the project. In the methods section, on pages six to seven in the first version of the article, we already explained why and how we used the IHC Key Concepts framework in the review. We do not think it is necessary to mention the authors’ involvement in IHC in the methods section. We have removed the last three paragraphs of the discussion, which were about IHC.

*The paper is long and complex – but with much valuable information. If any more material can be moved into Supp files, that will be helpful.*

 We agree that the article is long and complex, which follows the study being large and complex. There are already 10 supplementary files and six supplementary tables, and we do not think it would be helpful to have more. We appreciate that the article was a demanding read for the reviewers, however we do not expect many other people will read it start-to-finish. The findings are summarised in the abstract and “Key findings” section, and the implications are summarised in Table 5. Moreover, there will be a short, plain-language post on the F1000Research blog summarising the findings and discussing the implications. Although we include implications for other groups, the target audience for the article itself is primarily researchers. Communicating the findings and implications to other groups is a separate task. 

*Search strategy: It seems almost 2 years since it was updated. Authors might consider updating before indexing.*

 It is possible that there have been new, eligible studies since the last search, and we agree that an updated review could be of value, at some point. Others planning or reporting new studies may, indeed, want to conduct updated searches. However, that is beyond the scope of this review, which summarises relevant research up to August 2019.There are good reasons for ensuring that reviews of interventions, which inform healthcare decisions are up to date when they are published (and are kept up to date). However, this is a review of descriptive studies and we do not believe those reasons are relevant to this review. 

- *P5:  “We excluded inappropriate criteria…’   Perhaps “ineligible” might be better?*

We have replaced “ineligible [criteria]” with “inappropriate”, in the main text and Figure 1.

- *P7: Re: your sentences “Furthermore, we reframed negative quality criteria as positive, and paraphrased all quality criteria, for the data to be consistently framed and worded, and for the criteria to be succinctly labelled. For example, Bubela et al. report the number of news reports that “did not” specify whether a study was randomized, which was reframed as the number of news reports that did specify whether a study was randomized.”  *
*While this makes sense, I presume that sometimes that re-framing was not straight forward – and I wonder if there is anything more to say on any complexities associated with this approach. Also, if you can give a little more detail in the Bubela example, with numbers, that could help the reader.*

We found reframing the criteria straightforward, and there were few criteria that needed to be reframed as positive. As noted in the main text, each criterion included in the meta-analyses is presented in S10 File verbatim, as reported in the primary study, together with the reframed and paraphrased version that we used in the review. We have added numbers to the example with the criterion from the study by Bubela et al.

- *P7: “Risk of Bias”. Can the authors add a few words in text explaining why the "risk of bias" was solely about the risk of detection bias? A reader of an SR might normally expect a few different domains, so it would be good to just explain why this single domain was most relevant.*

We have added a paragraph about why we only assessed detection bias to the relevant section, with reference to the protocol and the section on deviations from the protocol, where we provide a detailed explanation.

- *P10: Table 2. I think Table 2 needs re-thinking – as it took me some time to work out what was going on. I was treating each row as a discreet coherent entity, but in fact you have multiple parts to each row. I am sure you can make it clearer somehow.*

We have asked the journal to change the formatting of Table 2 so that the columns are alternate colours, rather than the rows. 

- *P11: “In contrast, a checklist for people communicating evidence-based information about the effects of healthcare interventions includes the item “Help your audience to avoid misinterpreting continuous outcome measures,” granted the checklist is not for journalists.” I don’t fully understand meaning of this sentence - perhaps be a little clearer?*

Mentioning the item in the checklist was meant to emphasise that the concept is important for laypeople. Upon reconsideration, we have simply removed the sentence since it was unclear and inessential.

- *P12: This is a structural suggestion. By the sub-heading “Synthesis of Results” I am really wanting to hear some of the key findings – in terms of content (i.e. a strong compelling prose version of the data in Figure 3)  – but I feel I am still getting a lot of complex fine detail about the characteristics of studies and elements of them. I understand there is a lot to report – but some clear statements of the key findings – even summarised and abstracted a little – would be helpful around here to orient the reader.*

We have added a summary to the start of the “Synthesis of results” section, by moving up content from the “Key findings” section, like you suggest further down.

- *Also – a related point - I find the three paragraphs on P12 (and into P13) complex and hard to follow. I wonder whether this level of detail is necessary in the main text – and whether it might be moved to a supplementary – but that is for authors to decide. At the very least – if there is any way to make it all a little clearer around this point – that would be great for the reader. An example of the mention of the “ninth criteria” from Bubela and 12th from Haneef.  I think this is way too much detail for main text and authors run the risk of losing readers, for what is a very valuable SR.  *

Please see our response to your comment further up, about moving more material into supplementary files.

- *Now I have read further I realise you have a long section  - starting on P14 – on the results – as they relate to IHC concepts. After having read that very long section, I still feel the need for some kind of punchy summary of the key findings (e.g. harms, COIs, costs, other options not often mentioned…) somewhere in the results section.  This kind of summary doesn’t appear until start of Discussion – but I think could be included earlier.*

- *P18: I wonder if the dot points at bottom of P18 might be better in the Results – and help to drive the kind of summary I am suggesting above.*

We have added a summary to the start of the “Synthesis of results” section, by moving up content from the “Key findings” section, including the dot points.

- *Discussion: the paper feels like it morphs in the Discussion into a passionate advocacy for IHC materials – with a sometimes moralistic tone. I think the tone could be addressed. And I also think that in order to justify the IHC material in the Discussion, more needs to be said about IHC in the Introduction – and the authors relations with IHC need to be made more explicit in the Methods – as noted above.*

We have removed the last three paragraphs of the discussion, which were about IHC.

 Thank you again for the valuable feedback!
